# Supplementary material for: The nuclear pore complex prevents sister chromatid recombination during replicative senescence
Source: Nat Commun. 2020 Jan 9;11:160. doi: 10.1038/s41467-019-13979-5 (PMC6952416; doi:10.1038/s41467-019-13979-5)
Supplement: Supplementary file 1 — Supplementary Information [file 41467_2019_13979_MOESM1_ESM.pdf]

Supplementary Information

The nuclear pore complex prevents sister chromatid recombination during replicative senescence

Aguilera P. et al

Supplementary Fig. 1

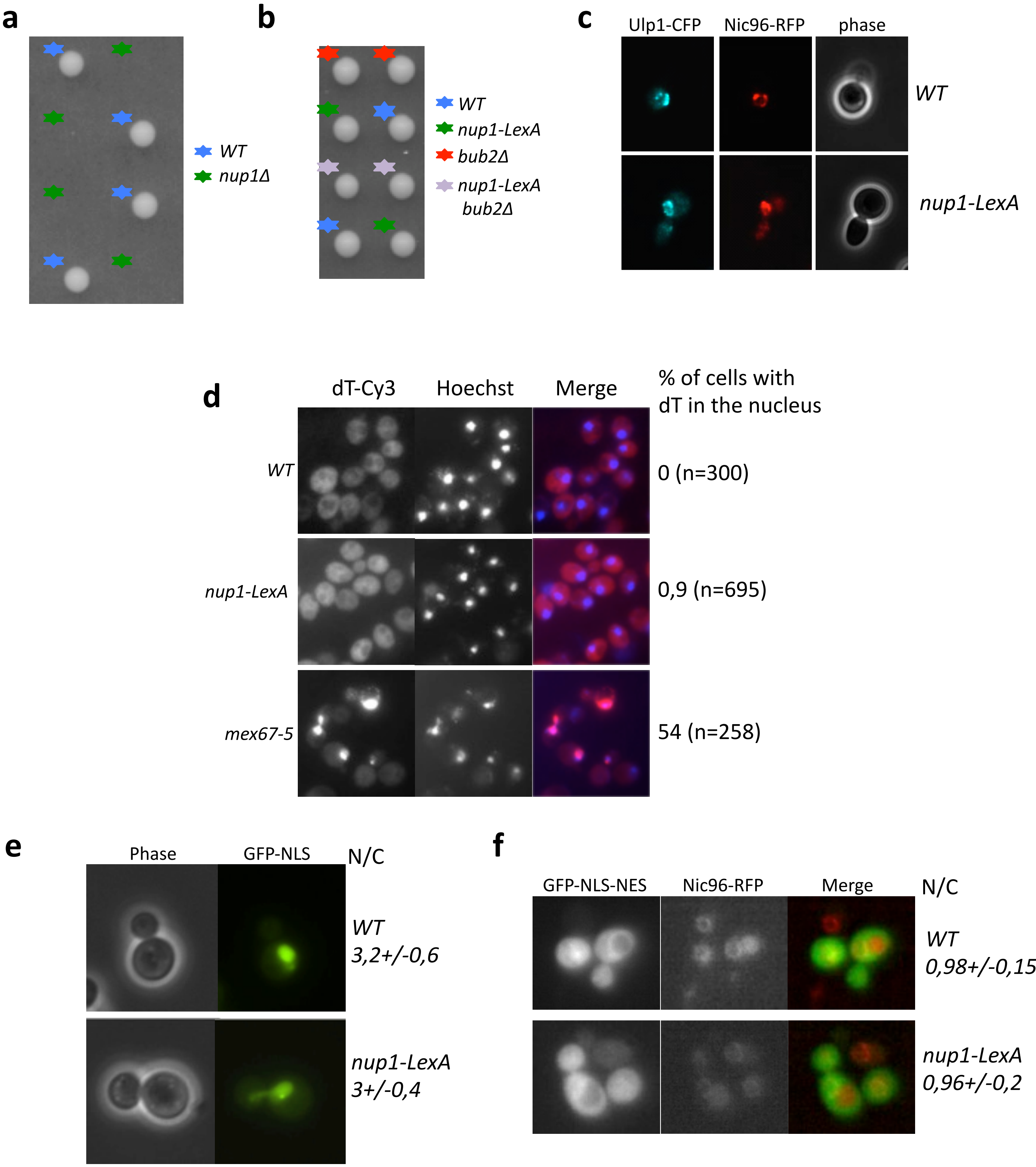

Supplementary Fig. 1

g

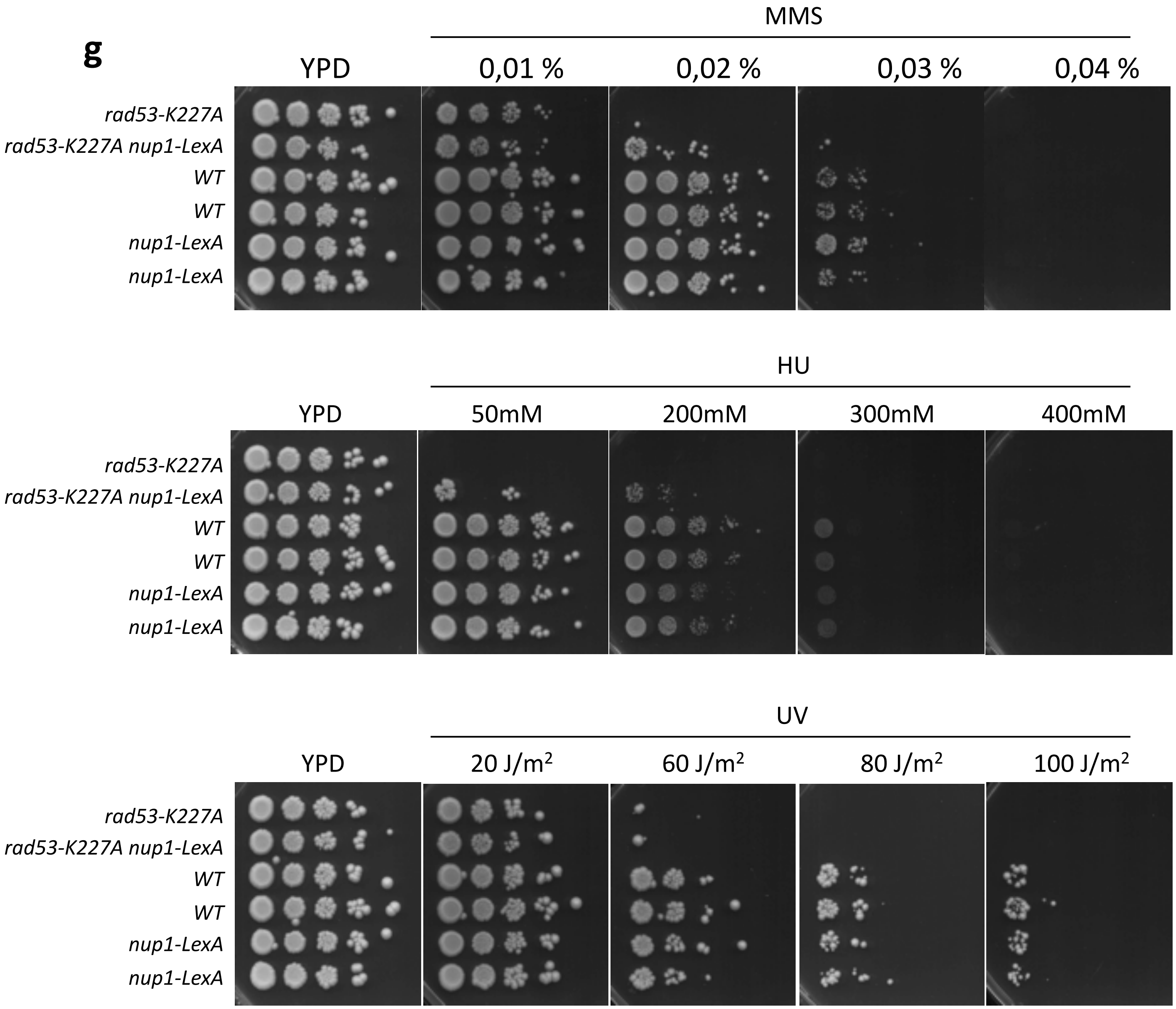

h

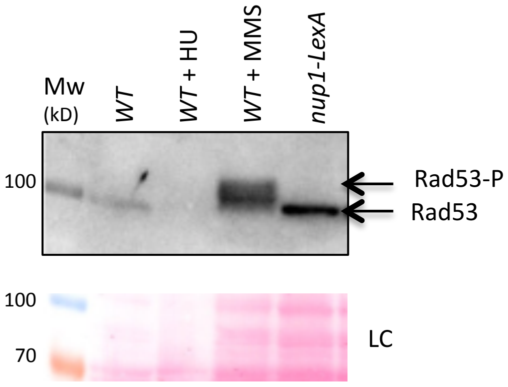

i

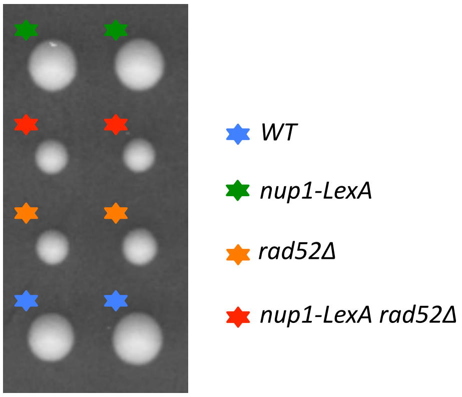

Supplementary Figure 1: Functionality of the Nup1-LexA fusion protein.

(a) *NUP1* deletion greatly affects growth in the W303 strain background. The *NUP1* gene was deleted in a WT diploid. After sporulation, meiotic products were analyzed after 3 days of growth at 30°C. **(b)** Meiotic products from *nup1-LexA bub2Δ* heterozygous diploid were analyzed by tetrad micromanipulation onto plates. Two representative tetrads are shown after 3 days of growth at 30°C. **(c)** WT and *nup1-LexA* cells expressing *ULP1-CFP* and *NIC96-RFP* were analyzed by fluorescence microscopy. Both fluorescence channels are shown along with the phase images of two representative cells. **(d)** Subcellular localization of poly(A)+ RNA was analyzed by FISH using a Cy3-labeled oligo-dT probe in WT (n=300) and nup1-LexA (n=395) cells as described[1]. The percentage of cells accumulating poly(A)+ RNA into the nucleus is shown. *mex67-5* mutant cells were used as a control. **(e)** Protein import was monitored with a GFP-NLS construct[2]. Fluorescence intensity was quantified in the nucleus (N) and cytoplasm (C) using ImageJ as described in methods, N/C ratios are indicated. **(f)** Protein export was monitored with a NES-GFP-NLS construct[2]. Fluorescence intensity was quantified in the nucleus (N) and cytoplasm (C) using ImageJ as described in methods, N/C ratios are indicated. **(g)** Ten-fold serial dilutions from stationary phase cultures of the indicated genotypes were plated onto YPD and YPD supplemented with increasing concentrations of either HU or MMS. The plates were incubated two days at 30°C. Four YPD plates were exposed to UV irradiation from 20 to 100J/m^2 before incubation. The experiment has been repeated three times with similar results. **(h)** *nup1-LexA* does not induce autophosphorylation of Rad53. WT and *nup1-LexA* cells were grown to mid-log phase and treated or not with HU (100mM) or MMS (0,1%) as indicated. Phosphorylation of Rad53 was visualized by western blotting using α-Rad53 antibodies. **(i)** Meiotic products from a *nup1-LexA rad52Δ* heterozygous diploid were analyzed by tetrad micromanipulation. Two representative tetrads out of nine analyzed are shown after 3 days at 30°C.

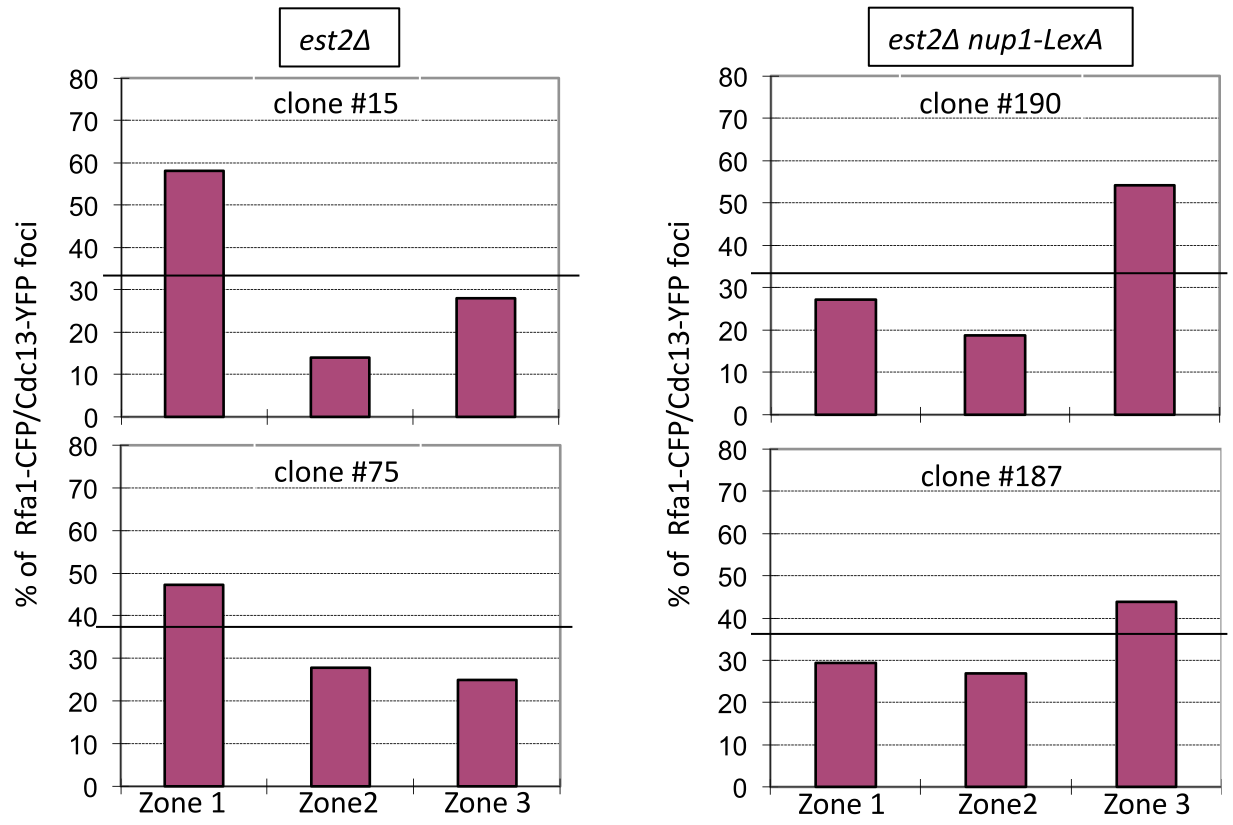

Supplementary Figure 2: *nup1-LexA* affects the localization of damaged telomeres.
Clonal analysis of the distribution of Cdc13-YFP/RfA1-CFP foci between three nuclear zones of equal area in late S and G2/M cells. Two independent clones were analyzed for each genotype.

a

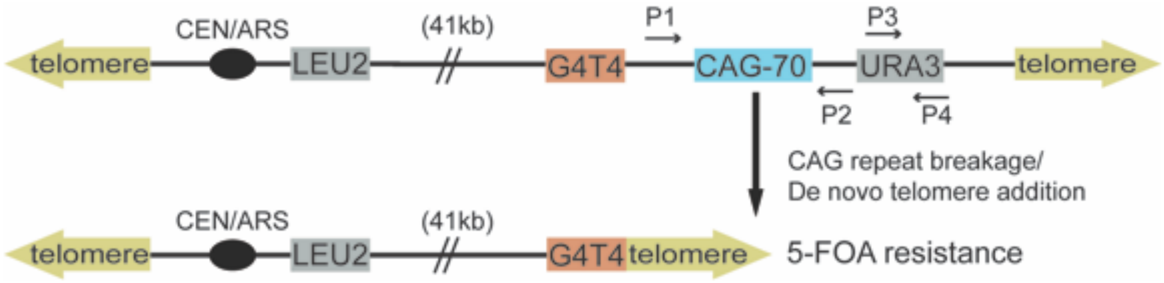

b

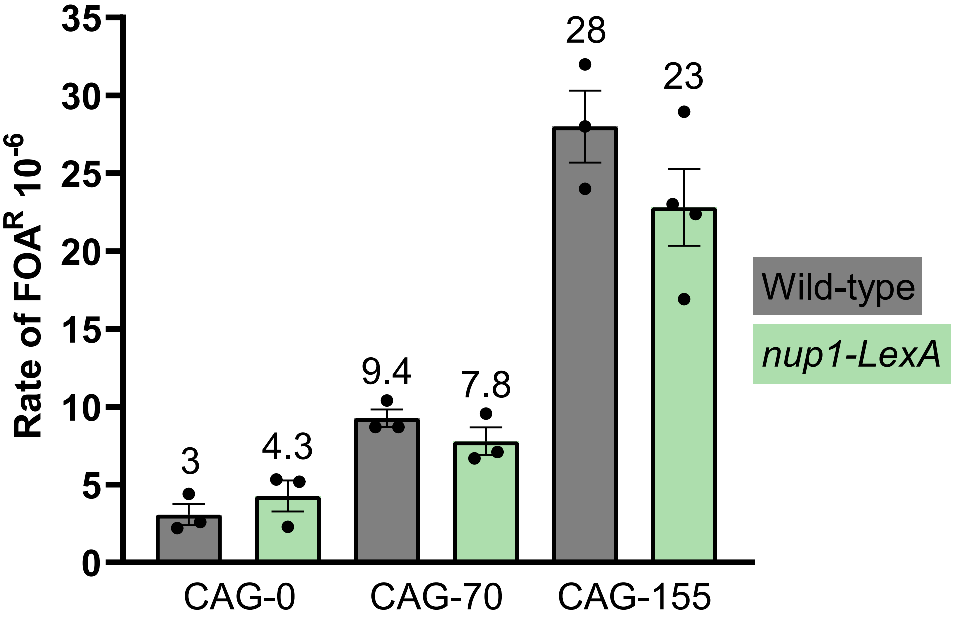

Supplementary Figure 3: Nup1-LexA does not affect fragility of CAG repeats.
(a) Schematic of the CAG fragility and instability assays. P1 and P2 arrows indicate primers used to confirm CAG tract length prior to each experiment or test instability frequency. P3 and P4 primers were used to check YAC end loss in FOAR colonies as described in the methods. The G4T4 sequence proximal to the CAG tract facilitates recovery of end loss events by providing a seed for telomere addition by telomerase. **(b)** Rate of FOAR x 10-6 in wild-type and *nup1-LexA* mutants for strains with YACs containing CAG-0, CAG-70, or CAG-155 repeats. The average of at least three experiments with standard error of the mean (SEM) is shown. For CAG-0 one experiment was done for strain #4216 and two for #4211. For CAG- 70 one experiment was done for strain #4217 and two for #4221. For CAG-155 three experiments were done for strain #4218. There were no significant differences between wildtype and *nup1-LexA* strains as determined by a Student's t-test.

Supplementary Fig. 4

a

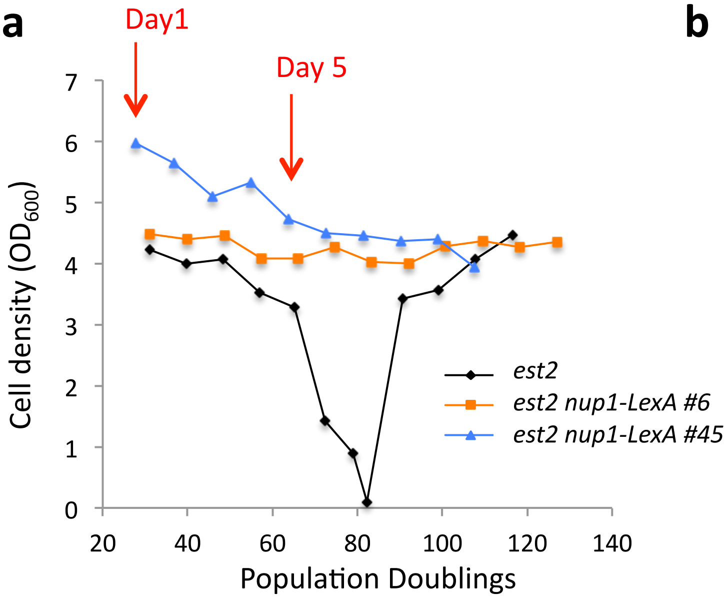

b

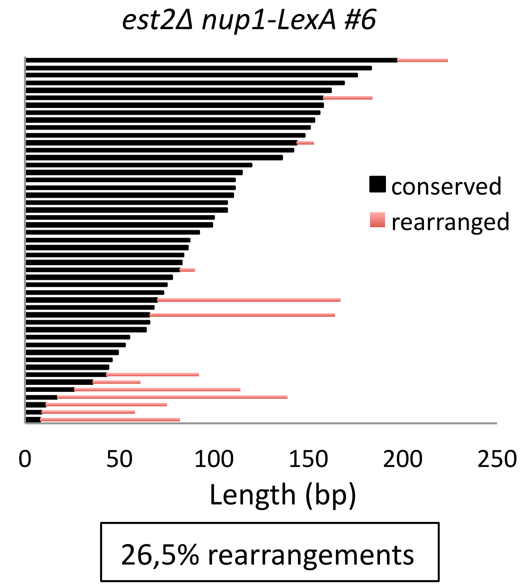

c

	est2Δ nup1-LexA #6 (26,5%)	*est2Δ nup1-LexA #A45 (28%)*
est2Δ (6%)	p=0,006	p=0,003

d

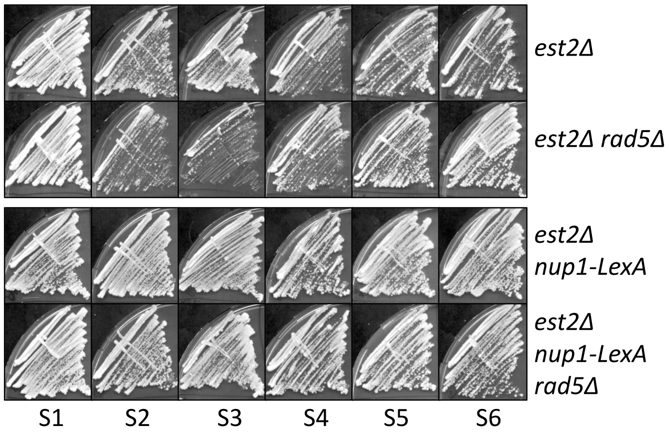

e

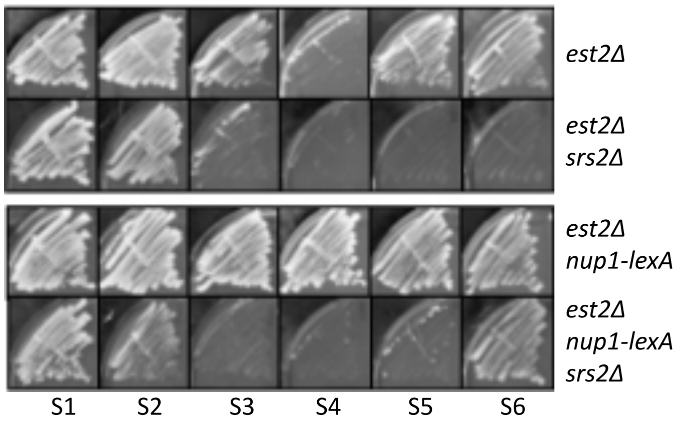

f

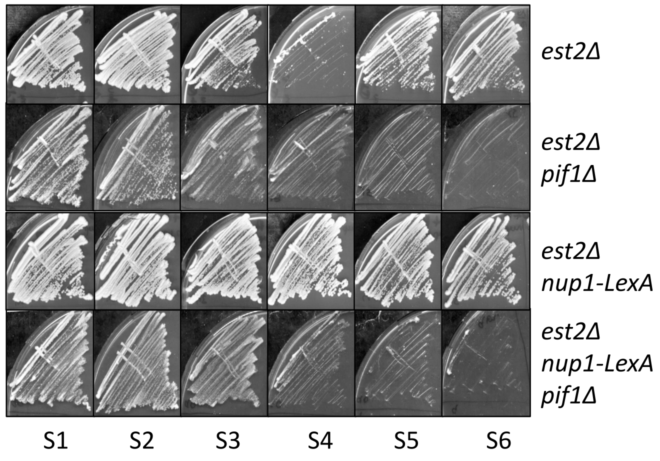

Supplementary Figure 4: Mechanism of telomere maintenance in *nup1-LexA* cells.

(a) Senescence curves of the three clones used for telomere sequencing. Cells from spore colonies were propagated in liquid culture through daily serial dilutions. OD_{600} was measured every day to estimate the cell density reached in 24 hours. PD numbers were estimated starting from the initial spores. **(b)** *TelVI-R* sequence analysis of the clone *est2Δ nup1-LexA #6* at D5 is shown. Each bar represents an individual telomere. Bars are sorted by the length of the centromere-proximal sequence that is identical to the reference sequence (black). The red bars show the length of the distal rearranged sequence that cannot be align to the reference sequence. **(c)** Pairwise p-values showing statistical significance of the difference in the frequency of *TelVI-R* rearrangements between *est2Δ* and *est2Δ nup1-LexA* clones #6 and #A45 (Fig. 3). P-values are from two-tailed X^2 test. **(d)** Growth capacity of representative clones of *est2Δ, est2Δ rad5Δ, est2Δ nup1-LexA* and *est2Δ nup1-LexA rad5Δ*. The first streak (S1) was inoculated from the initial spore colony and restreaked on a second plate (S2) after 2 days at 30°C. Each clone is then restreaked on a new plate and grown for 2 days. Each panel is representative of three to six independent clones of the same genotype. **(e)** Growth capacity of representative clones of *est2Δ, est2Δ srs2Δ, est2Δ nup1-LexA* and *est2Δ nup1-LexA srs2Δ* on solid medium. Restreaks were performed as detailed above. **(f)** Growth capacity of representative clones of *est2Δ, est2Δ pif1Δ, est2Δ nup1-LexA* and *est2Δ nup1-LexA pif1Δ* on solid medium.

Supplementary Fig. 5

a

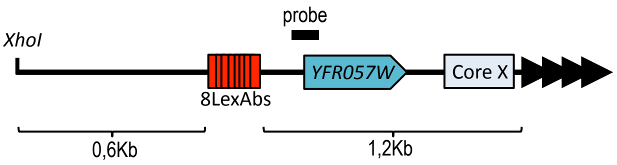

b

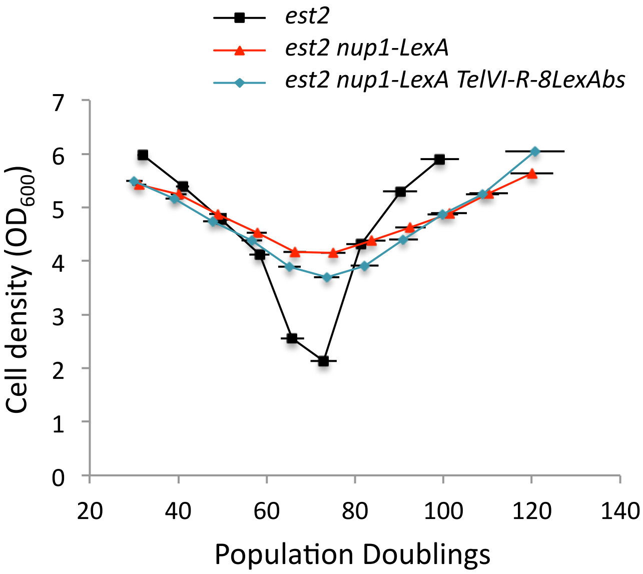

c

	est2Δ nup1-LexA TelVIR-8LexAbs #15 (16%)	*est2Δ nup1-LexA TelVIR-8LexAbs #A68 (9%)*
est2Δ (6%)	p=0,11	p=0,57
est2Δ nup1-LexA #6 (26,5%)	p=0,22	P=0,011
est2Δ nup1-LexA #A45 (28%)	P=0,15	P=0,005

d

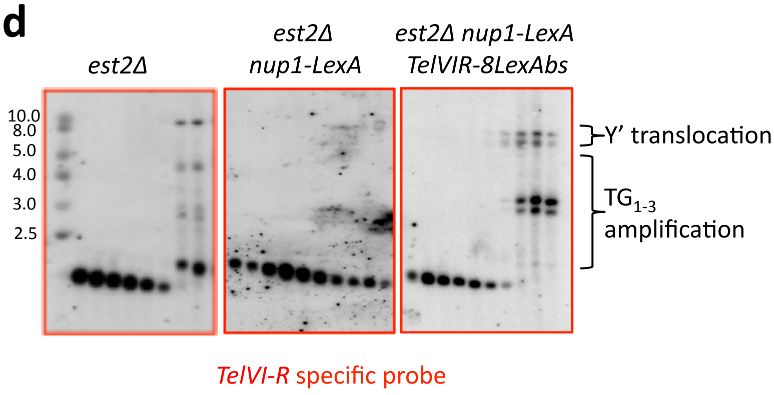

e

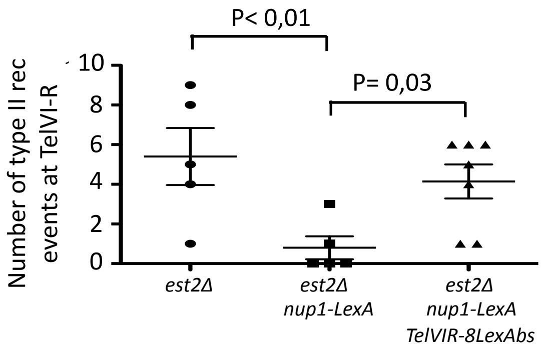

Supplementary Figure 5: Tethering of *TelVI-R* to the NPC partially rescues the phenotype of *est2Δ nup1-LexA* cells.

(a) Shematic of the modified *TelVI-R-8LexAbs* telomere*.* The site of insertion of the LexA DNA binding sites and the position of the specific *TelVI-R probe* are shown. **(b)** Mean senescence profiles of *est2Δ nup1-LexA TelVI-R-8LexAbs* clones (n=9). The senescence profiles of *est2Δ* and *est2Δ nup1-LexA* cells are from Fig. 1 and are reproduced here for comparison. Error bars are SD. **(c)** Pairwise p-values showing statistical significance of the difference in the frequency of *TelVI-R* rearrangements. P-values are from two-tailed X^2 test. **(d)** The effect of tethering *TelVI-R* to the NPC was evaluated by Southern blot of *XhoI* cut DNA revealed with the *TelVI-R* specific probe shown in **(a)**. The results are shown for one representative clone of each genotype. **(e)** Quantification of the effect of telomere VI-R targeting on the frequency of type II recombination events. The number of type II recombination events in each clonal cell population was counted from the corresponding Southern blot probed with a specific *TelVI-R* fragment at the last time point of survivor formation. Mean values are plotted for *est2Δ* (n=5 clones)*, est2Δ nup1-LexA* (n= 5 clones) and *est2Δ nup1-LexA TelVI-R-8LexAbs* (n=7 clones). The error bars represent SEM. P-values are from Fisher's least significant difference (LSD) test.

a

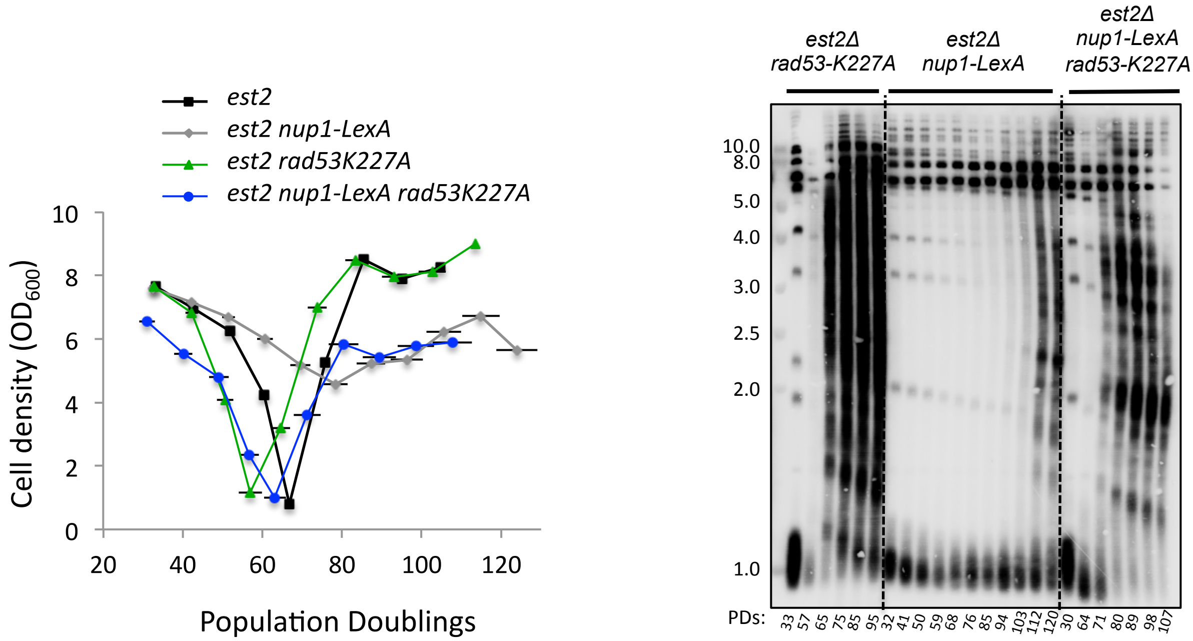

b

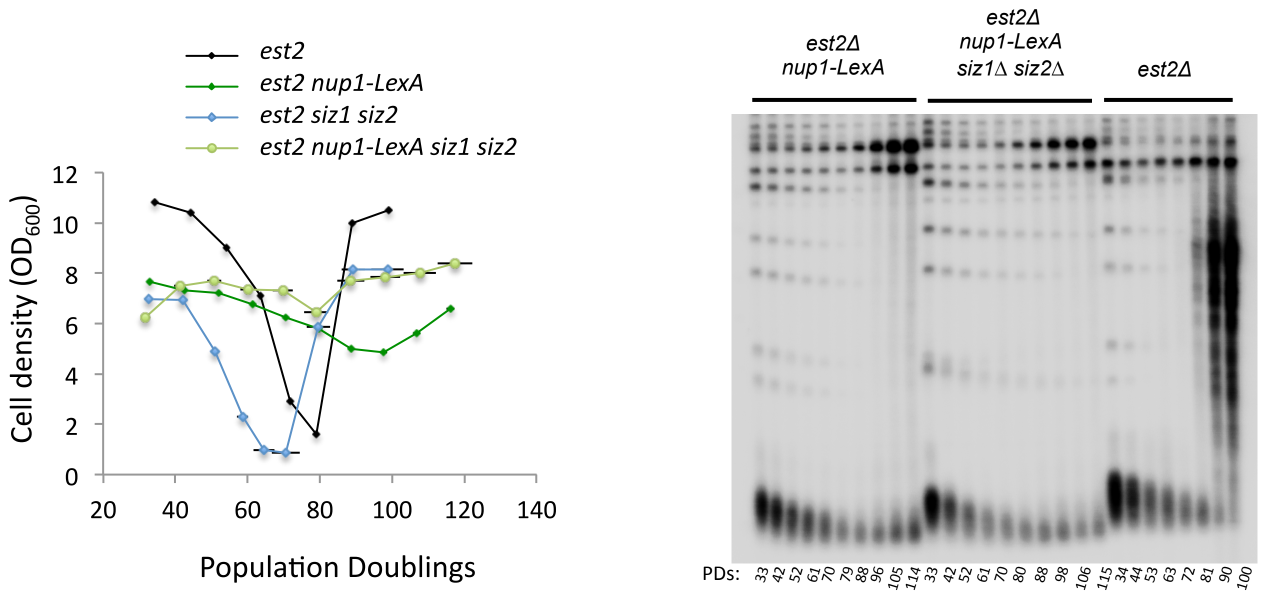

Supplementary Figure 6: The growth rate of *nup1-LexA est2Δ* cells depends on the integrity of the DNA damage checkpoint protein Rad53^{CHK2} but not on Siz1, Siz2-dependent SUMOylation.

(a) Mean senescence profiles of *est2Δ* (n=2), *est2Δ rad53-K227A* (n=5), *est2Δ nup1-LexA* (n=4) and *est2Δ nup1-LexA rad53-K227A* (n=4) clones. Error bars are SD. Southern blot of *XhoI*-digested DNA prepared from samples of senescing cells was revealed with a $TG_{1\text{-}3}$ probe. One representative clone of each genotype is shown. **(b)** Mean senescence profiles of *est2Δ siz1Δ siz2Δ* (n=3), *est2Δ nup1-LexA* (n=4) and *est2Δ nup1-LexA siz1Δ siz2Δ* (n=4) clones along with one *est2Δ* clone. Error bars are SD. Telomere length analysis of three representative clones with the indicated genotypes is shown. Southern blot of *XhoI*-digested DNA was revealed with a $TG_{1\text{-}3}$ probe.

Supplementary Fig. 7

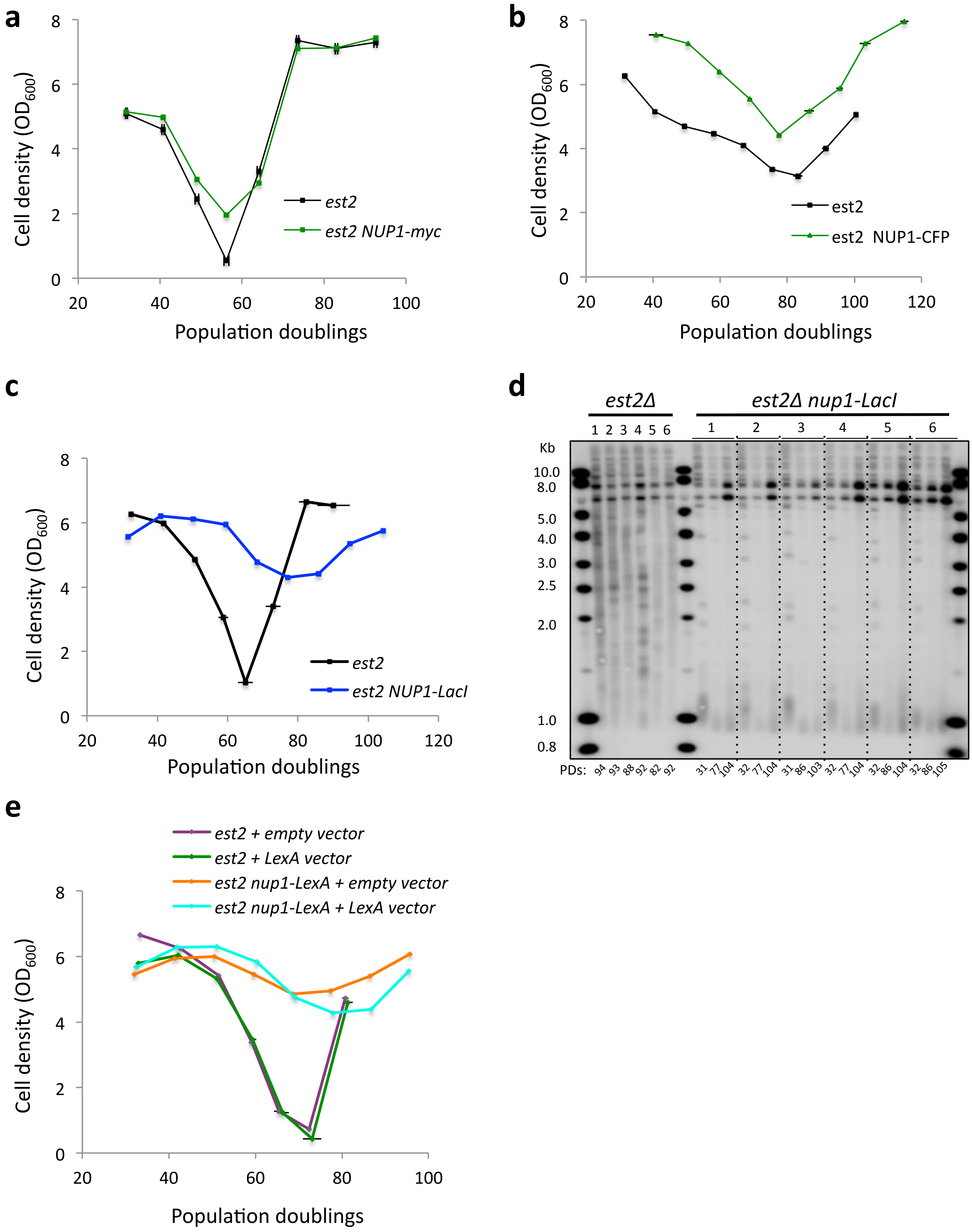

Supplementary Figure 7: The LexA DNA-binding moiety is required for continuous growth of *est2Δ nup1-LexA* cells.

(a) Mean senescence profile of *est2Δ NUP1-Myc*$_{13}$ clones (n=4) as compared to *est2Δ* clones (n=2) isolated from the same diploid strain. Error bars are SD. **(b)** Mean senescence profile of *est2Δ NUP1-CFP* (n=5) as compared to *est2Δ* (n=4) clones isolated from the same diploid strain. **(c)** Mean senescence profile of *est2Δ nup1-LacI* (n=6) as compared to *est2Δ* (n=6) clones isolated from the same diploid strains. Two heterozygous *est2Δ nup1-LacI* diploid strains were used in this experiment that were generated from two independently isolated *nup1-LacI* clones. Error bars are SD. **(d)** Telomere length and survivor formation of the clones shown in (b) were monitored by $TG_{1\text{-}3}$ probed Southern blots of *XhoI*-digested DNA. Only the last time points are shown for the *est2Δ* control clones and three time points (one day of growth, peak of senescence and last point after recovery) for the *est2Δ NUP1-LacI* clones. **(e)** Mean senescence profile of *est2Δ* and *est2Δ nup1-LexA* clones carrying a empty vector (YepLac195) or a vector expressing LexA (pBTM116-URA3[3]). Both vectors are multicopy 2μ vectors. LexA is expressed under the *ADH1* promoter. *est2Δ* + empty vector (n=4), *est2Δ* + LexA (n=5), *est2Δ nup1-LexA* + empty vector (n=5), *est2Δ nup1-LexA* + LexA vector (n=6). Error bars are SD.

a

	Zone 1 foci		Zone 2 foci		Zone 3 foci		Total # of cells
	%	#	%	#	%	#	
**Wild-type**	53	51	21	20	26	25	96
**NUP1-LexA**	28	33	22	27	50	60	120
nup1ΔCt	32	45	29	40	39	54	139

b

	Zone 1 foci		Zone 2 foci		Zone 3 foci		Total # of cells
	%	#	%	#	%	#	
**Wild-type**	47	128	31	84	22	61	273
**NUP1-LexA**	30	43	20	28	50	71	142
nup1ΔCt	29	42	16	22	55	79	143

Supplementary Figure 8: *nup1ΔCt* affects the localization of damaged telomeres and CAG repeats. a) analysis of the distribution of Cdc13-YFP/RfA1-CFP foci between three nuclear zones of equal area in *est2Δ, est2Δnup1-LexA and est2Δ nup1ΔCt* cells in late S and G2/M. b) The percent and number of zone 1, zone 2, and zone 3 foci for CAG-130 S-phase cells is shown for Wild-type, *nup1-LexA*, and *nup1ΔCt*. The total number of cells analyzed per strain ranges from 142-273.

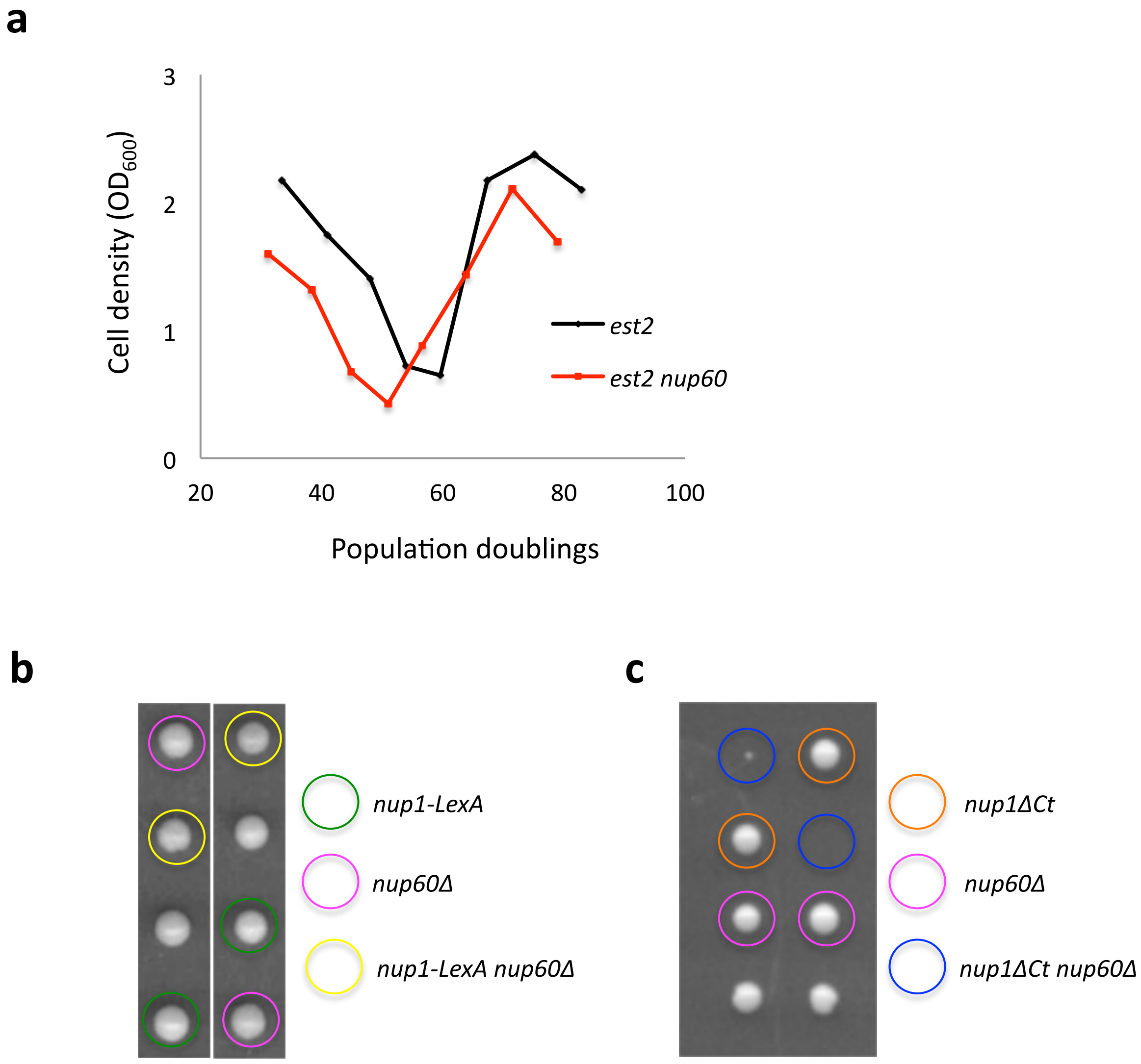

Supplementary Figure 9: *nup1-LexA* cells do not show genetic interaction with the deletion of *NUP60*.

(a) Mean senescence profile of *est2Δ* (n=4) as compared to *est2Δ nup60Δ* (n=4) clones isolated from the same diploid strain. Error bars are SD. **(b)** Meiotic products from *nup1-LexA nup60Δ* heterozygous diploid were analyzed by tetrad micromanipulation onto plates. The spore colonies from two representative tetrads are shown after 3 days of growth at 30°C. **(c)** Meiotic products from *nup1ΔCt nup60Δ* heterozygous diploid were analyzed by tetrad micromanipulation onto plates. The spore colonies from two representative tetrads are shown after 3 days of growth at 30°C.

Strain name	Genotype	Origin
W303-1A	*MATα ade2-1 can1-100 his3-11,15 leu2-3-112 trp1-1 ura3 rad5-G535R*	
W1588	*MATα ade2-1 can1-100 his3-11,15 leu2-3-112 trp1-1 ura3 RAD5*	
MNY1372	*MATα nup1-LexA::TRP1*	This study
MNY1373	*nup1-LexA::TRP1 TELVI-R::8LexABS-LoxP*	This study
MNY1363	*MATa/ MATα EST2/ est2::LEU2 NUP1/ nup1-LexA::TRP1 TELVI-R/ TELVI-R::8LexABS-LoxP*	This study
MNY1381	*MATa/ MATα EST2/ est2::kanMX6 NUP1/ nup1-LexA::TRP1 RAD51/ rad51::LEU2*	This study
MNY1388	*MATa/ MATα EST2/ est2::LEU2 NUP1/ nup1-LexA::TRP1 RAD59/ rad59::kanMX6*	This study
MNY1429	*MATa rad5::TRP1*	This study
MNY1431	*MATa/ MATα EST2/ est2::kanMX6 NUP1/ nup1-LexA::TRP1 RAD5/ rad5::TRP1*	This study
ML78-4A	*MATα srs2::HIS3*	M. Lisby
MNY1460	*MATa/ MATα EST2/ est2::kanMX6 NUP1/ nup1-LexA::TRP1 SRS2/ srs2 ::HIS3*	This study
MNY1406	*MATa/ MATα EST2/ est2::LEU2 NUP1/NUP1-CFP::URA3*	This study
MNY1449	*MATa/ MATα EST2/ est2::LEU2 NUP1/ nup1-LexA::TRP1 RAD53/ rad53-K227A::kanMX6*	This study
MNY1356	*nup60::kanMX6*	J. Drogbat
ML302	*MATα NIC96-RFP::kanMX6 ADE+*	M. Lisby
MNY1379	*NUP1-lexA::TRP1 NIC96-RFP::kanMX6*	This study
MNY1410	*NIC96-RFP::kanMX6 ULP1-CFP::URA3*	This study
MNY1411	*nup1-LexA::TRP1 NIC96-RFP::kanMX6 ULP1-CFP::URA3*	This study
JH77	*MATa rad52::kanMX6*	ref[4]
MNY1398	*MATa/ MATα EST2/ est2::LEU2 CDC13/CDC13-YFP RFA1/RFA1-CFP NUP1/nup1-LexA::TRP1 NIC96/NIC96-RFP::kanMX6*	This study
MNY1585	*MATa/ MATα EST2/est2::kanMX6 NUP1-LacI::TRP1 pEST2::URA3*	This study
MNY1587	*MATa/ MATα EST2/est2::kanMX6 NUP1-LacI::TRP1 pEST2::URA3*	This study
CHF765	*Wild-type BY4705 + YAC CF1 (ade3-2p, LEU2,* (CAG/CTG)0 *URA3)*	ref[5]
CHF766	*Wild-type BY4705 + YAC CF1(ade3-2p, LEU2,* (CAG/CTG)70 *URA3)*	ref[5]

Strain name	Genotype	Origin
CHF767	*Wild-type BY4705 + YAC CF1(ade3-2p, LEU2,* (CAG/CTG)155 *URA3)*	ref[5]
CHF1819	*CHF766 rad52::HIS3 CAG-70*	ref[6]
CHF1820	*CHF766 rad52::HIS3 CAG-70*	ref[6]
CHF4211	*CHF765 nup1-LexA::TRP1 CAG-0*	This study
CHF4216	*CHF765 nup1-LexA::TRP1 CAG-0*	This study
CHF4217	*CHF766 nup1-LexA ::TRP1 CAG-70*	This study
CHF4221	*CHF766 nup1-LexA::TRP1 CAG-70*	This study
CHF4218	*CHF767 nup1-LexA::TRP1 CAG-155*	This study
CHF4459	CHF4217 *nup1-LexA::TRP1 rad52::NATMX CAG-70*	This study
CHF4460	CHF4217 *nup1-LexA::TRP1 rad52::NATMX CAG-70*	This study
CHF2744	*MATa ade2-1 can1-100 his3-11,-15::GFP-LacI:HIS3 trp1-1, ura3-1, leu2-3,-112 nup49::GFP-NUP49 Chr6int1::lacO:4xLexA array:TRP1 Chr6int2::CAG130:HPH (Chr6int2* is 6371bp from *Chr6int1* and *560bp* upstream of *Ta(AGC)F* gene)	ref[6]
CHF4469	*CHF2744 nup1-LexA::kanMX6*	This study
CHF4470	*CHF2744 nup1-LexA::kanMX6*	This study
CHF4909	*CHF2744 nup1ΔCt::kanMX6*	This study
CHF4910	*CHF2744 nup1ΔCt::kanMX6*	This study
2062	*nup1ΔFxFG*	ref[8]
MNY1651	*MATa/ MATα EST2/est2::LEU2 NUP1/nup1ΔFxFG pEST2::URA3*	This study
MNY1670	*MATa nup1ΔCt::TRP1*	This study
MNY1652	*MATa/ MATα EST2/est2::LEU2 NUP1/nup1ΔCt::TRP1 pEST2::URA3*	This study
MNY1673	*MATa/ MATα EST2/ est2::LEU2 CDC13/CDC13-YFP RFA1/RFA1-CFP NUP1/nup1ΔCt::TRP1 NIC96/NIC96-RFP::kanMX6*	This study<br>This study
MNY1685	*MATa/ MATα EST2/est2::LEU2 NUP1/nup1-LexA::TRP1 siz1::kanMX6 siz2::kanMX6 pEST2::URA3*<br>*MATα ade2-1 can1-100 his3-11,15 leu2-3-112 trp1-1 ura3 rad5-G535R*	

Supplementary Table 1: Yeast strains used in this study.

Strains are derivatives of W303-1A (*MAT***a** *BAR1 LYS2 ade2-1 can1-100 ura3-1 his3-11,15 leu2-3, 112 trp1-1 rad5-535*)[7] or it's derivative W1588 (*MAT***a** *BAR1 LYS2 ade2-1 can1-100 ura3-1 his3-11,15 leu2-3, 112 trp1-1 RAD5*).). CHF CAG-0, CAG-70, and CAG-155 strains used for instability and fragility analysis contain the CAG tract on YAC CF1, and are derivatives of CHF765, 766, or 767 in the BY4705 background (*MATα, ade2Δ::hisG, his3Δ200, leu2Δ0, lys2Δ0, met15Δ0, trp1Δ63, ura3Δ0,* can^R*, YAC: ade3-2p, LEU2,* $(CAG/CTG)_n$ *URA3)*. CAG-130 strains used in the zoning analysis were derivatives of CHF2744 (W303 background). For all CAG/CTG-containing strains, the tract is oriented such that the CAG sequence is on the lagging strand template.

Supplementary References :

1. Babour, A. *et al*. The Chromatin Remodeler ISW1 Is a Quality Control Factor that Surveys Nuclear mRNP Biogenesis. *Cell 167*, 1201-1214.
2. Nino, C.A. *et al.* Posttranslational marks control architectural and functional plasticity of the nuclear pore complex basket. *J Cell Biol* **212**, 167-180 (2016).
3. Texari, L. *et al.* The nuclear pore regulates GAL1 gene transcription by controlling the localization of the SUMO protease Ulp1. *Mol Cell* **51**, 807-818 (2013).
4. Texari, L. *et al.* The nuclear pore regulates GAL1 gene transcription by controlling the localization of the SUMO protease Ulp1. *Mol Cell* **51**, 807-818 (2013).
5. Sundararajan, R., Gellon, L., Zunder, R.M. & Freudenreich, C.H. Double-strand break repair pathways protect against CAG/CTG repeat expansions, contractions and repeat-mediated chromosomal fragility in Saccharomyces cerevisiae. *Genetics* **184**, 65-77 (2010).
6. Su, X.A., Dion, V., Gasser, S.M. & Freudenreich, C.H. Regulation of recombination at yeast nuclear pores controls repair and triplet repeat stability. *Genes Dev* **29**, 1006-1017 (2015).
7. Thomas, B.J. & Rothstein, R. Elevated recombination rates in transcriptionally active DNA. *Cell* **56**, 619-630 (1989).
8. Strawn, L.A., Shen, T., Shulga, N., Goldfarb, D.S. & Wente, S.R. Minimal nuclear pore complexes define FG repeat domains essential for transport. *Nat Cell Biol* **6**, 197-206 (2004).
