# Supplementary material for: The nuclear pore complex prevents sister chromatid recombination during replicative senescence
Source: Nat Commun. 2020 Jan 9;11:160. doi: 10.1038/s41467-019-13979-5 (PMC6952416; doi:10.1038/s41467-019-13979-5)
Supplement: Supplementary file 4 — Source Data [file 41467_2019_13979_MOESM4_ESM.zip › Source Data - Unprocessed Scans.pdf]

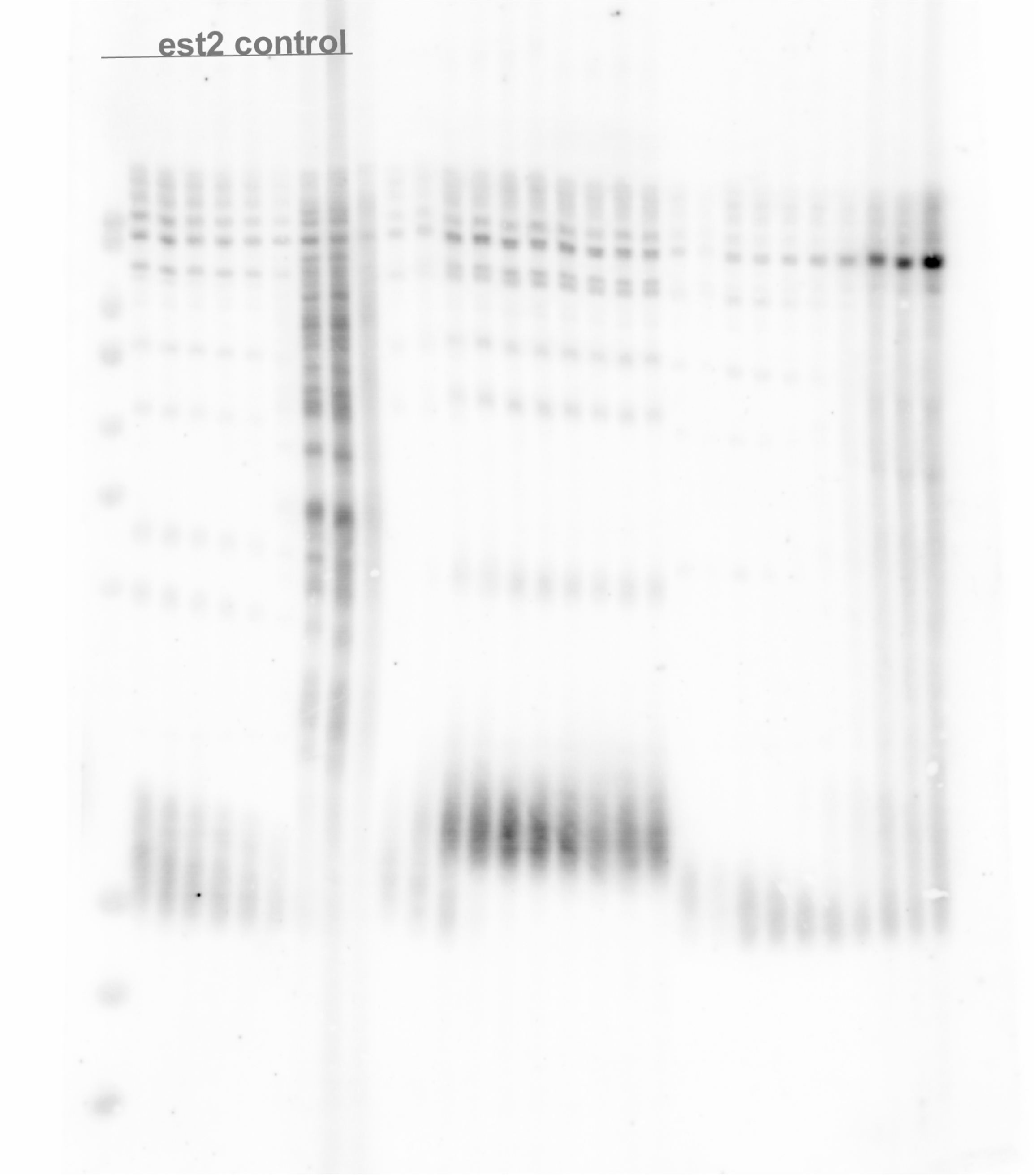
est2 control

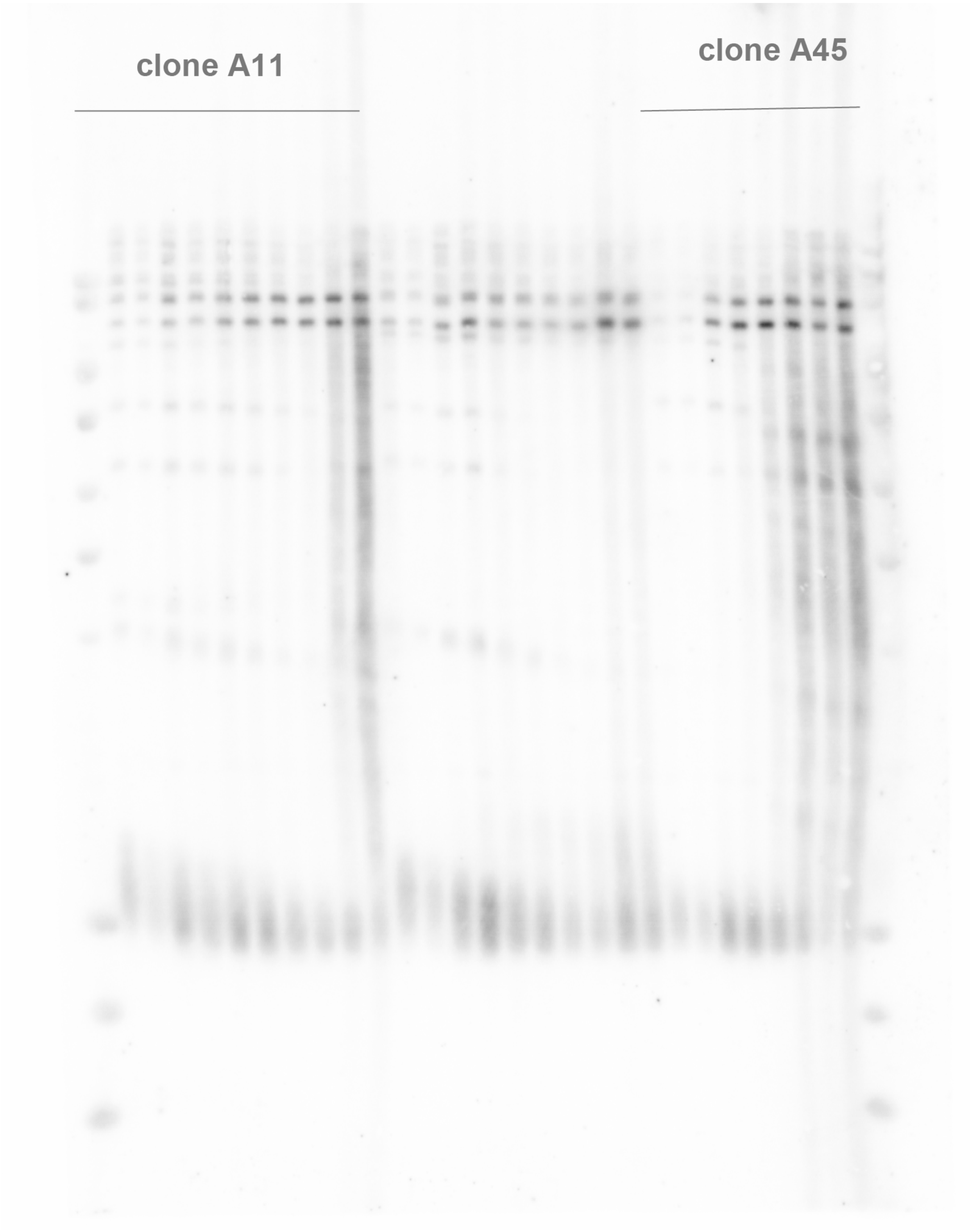
clone A11
clone A45

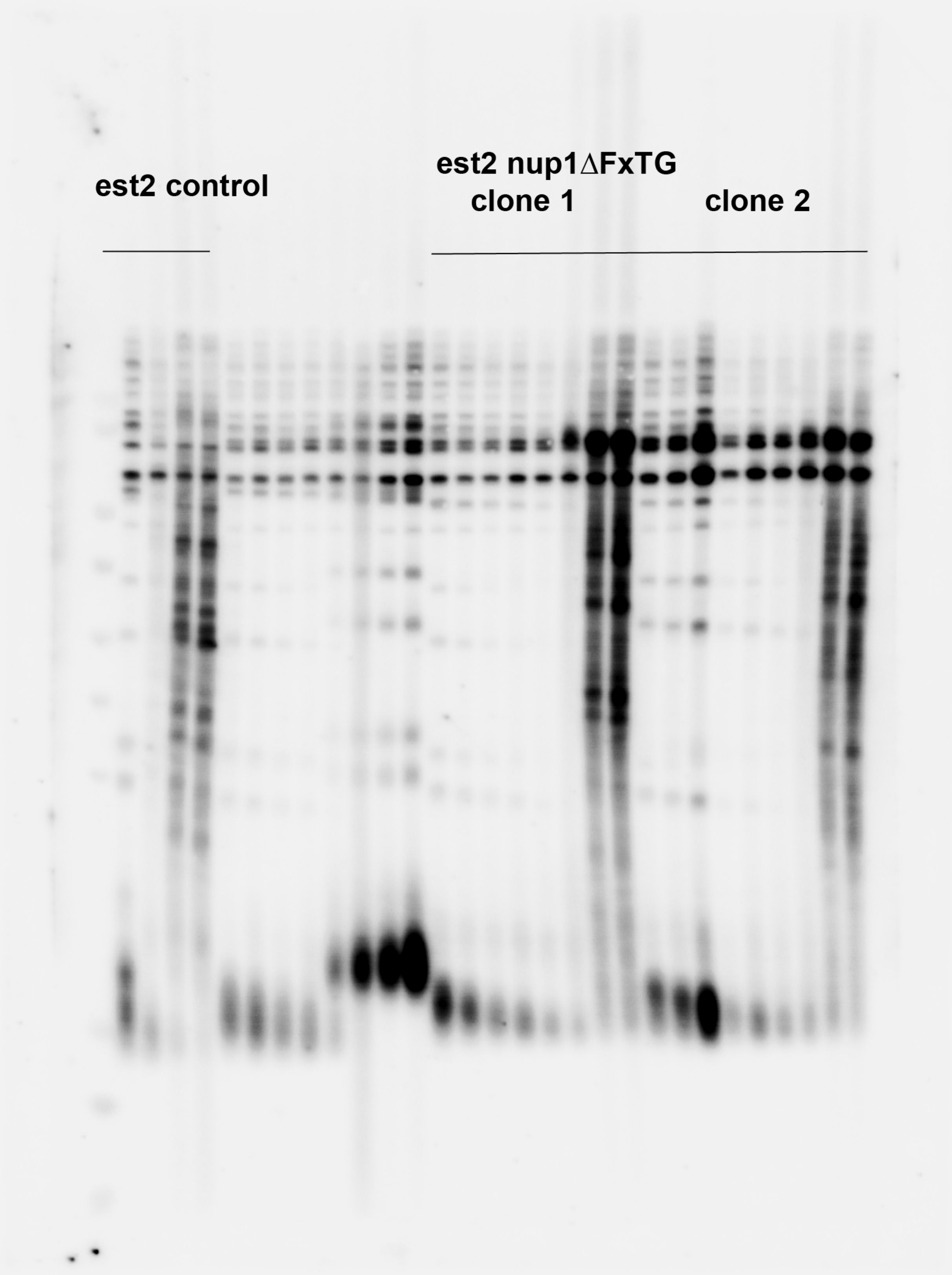
est2 control
est2 nup1∆FxTG
clone 1
clone 2

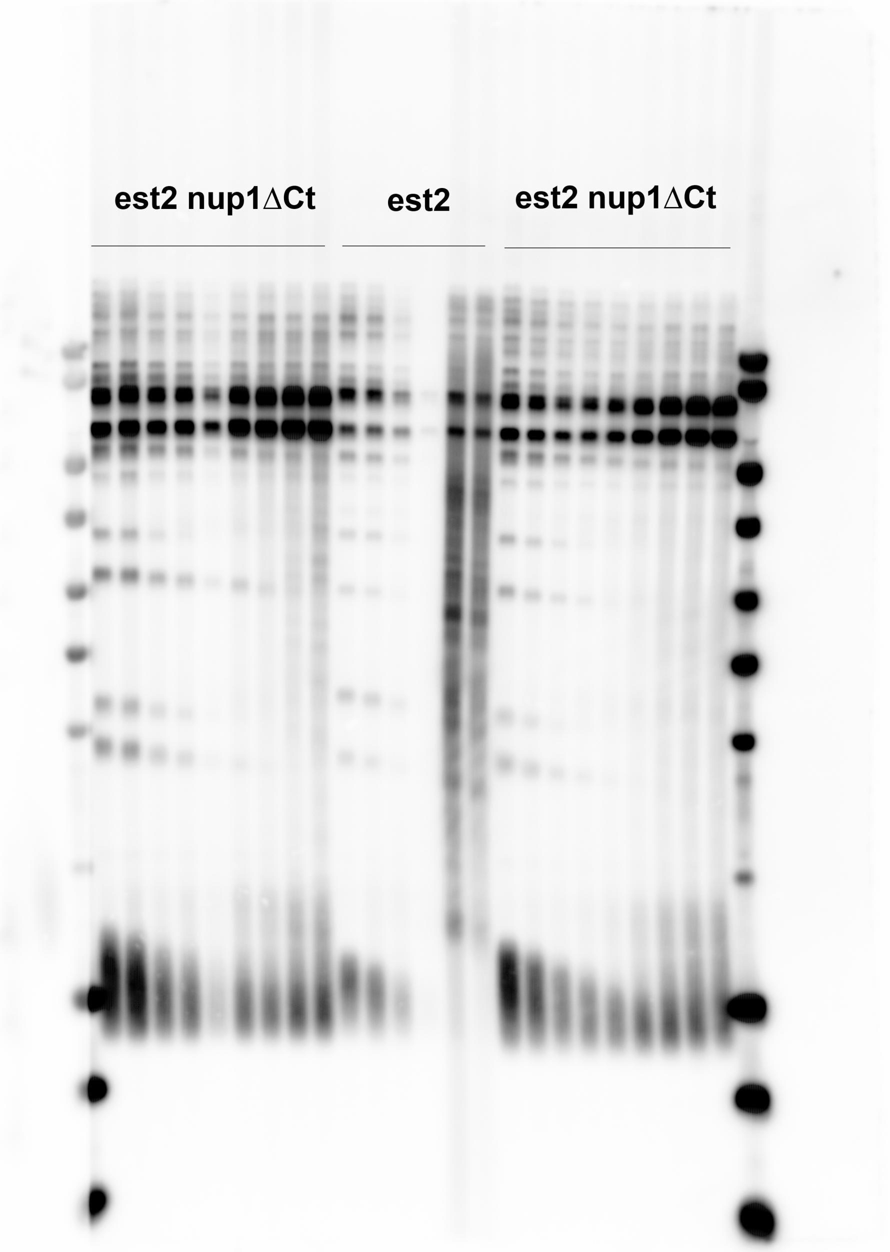
est2 nup1ΔCt
est2
est2 nup1ΔCt
