# Supplementary material for: The nuclear pore complex prevents sister chromatid recombination during replicative senescence
Source: Nat Commun. 2020 Jan 9;11:160. doi: 10.1038/s41467-019-13979-5 (PMC6952416; doi:10.1038/s41467-019-13979-5)
Supplement: Supplementary file 4 — Source Data [file 41467_2019_13979_MOESM4_ESM.zip › Source Data - Individual Senescence Curves.pptx]

## Slide 1
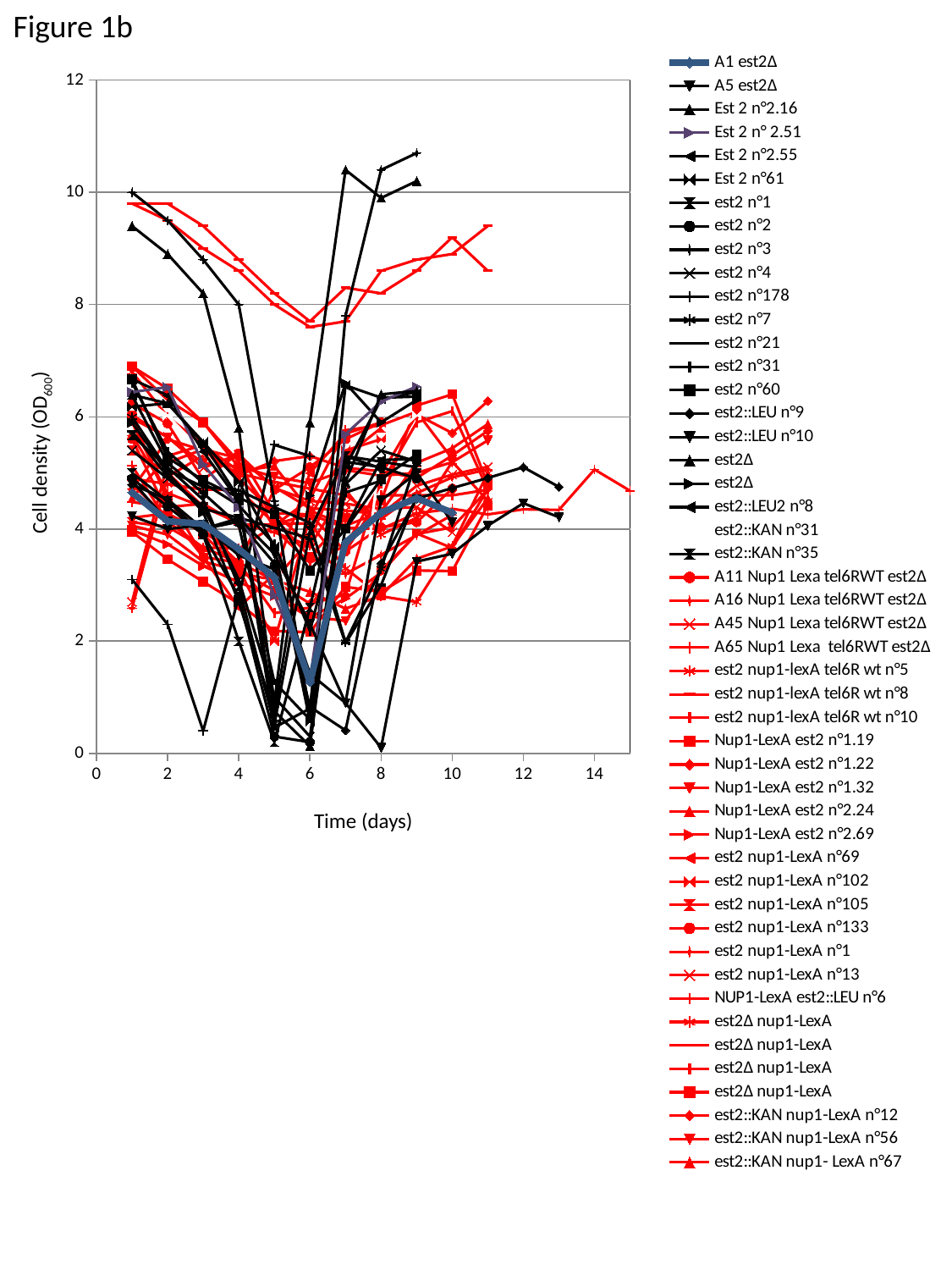

Figure 1b
### Chart
| Category | A1 est2∆ | A5 est2∆ | Est 2 n°2.16 | Est 2 n° 2.51 | Est 2 n°2.55 | Est 2 n°61 | est2 n°1 | est2 n°2 | est2 n°3 | est2 n°4 | est2 n°178 | est2 n°7 | est2 n°21 | est2 n°31 | est2 n°60 | est2::LEU n°9 | est2::LEU n°10 | est2Δ | est2Δ | est2::LEU2 n°8 | est2::KAN n°31 | est2::KAN n°35 | A11 Nup1 Lexa tel6RWT est2∆ | A16 Nup1 Lexa tel6RWT est2∆ | A45 Nup1 Lexa tel6RWT est2∆ | A65 Nup1 Lexa tel6RWT est2∆ | est2 nup1-lexA tel6R wt n°5 | est2 nup1-lexA tel6R wt n°8 | est2 nup1-lexA tel6R wt n°10 | Nup1-LexA est2 n°1.19 | Nup1-LexA est2 n°1.22 | Nup1-LexA est2 n°1.32 | Nup1-LexA est2 n°2.24 | Nup1-LexA est2 n°2.69 | est2 nup1-LexA n°69 | est2 nup1-LexA n°102 | est2 nup1-LexA n°105 | est2 nup1-LexA n°133 | est2 nup1-LexA n°1 | est2 nup1-LexA n°13 | NUP1-LexA est2::LEU n°6 | est2Δ nup1-LexA | est2Δ nup1-LexA | est2Δ nup1-LexA | est2Δ nup1-LexA | est2::KAN nup1-LexA n°12 | est2::KAN nup1-LexA n°56 | est2::KAN nup1- LexA n°67 |
|---|---|---|---|---|---|---|---|---|---|---|---|---|---|---|---|---|---|---|---|---|---|---|---|---|---|---|---|---|---|---|---|---|---|---|---|---|---|---|---|---|---|---|---|---|---|---|---|---|Cell density (OD600)
Time (days)

## Slide 2
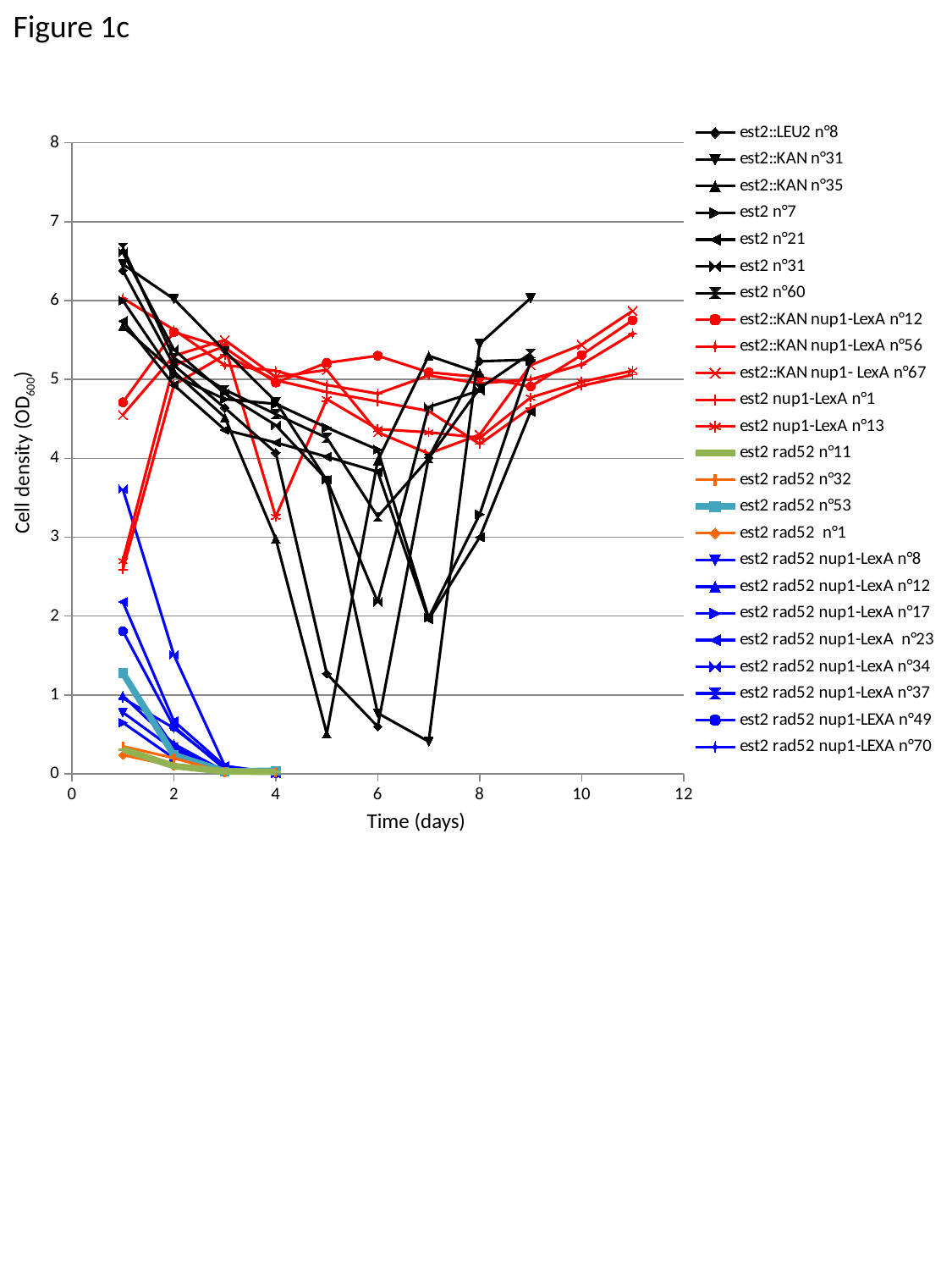

Figure 1c
### Chart
| Category | est2::LEU2 n°8 | est2::KAN n°31 | est2::KAN n°35 | est2 n°7 | est2 n°21 | est2 n°31 | est2 n°60 | est2::KAN nup1-LexA n°12 | est2::KAN nup1-LexA n°56 | est2::KAN nup1- LexA n°67 | est2 nup1-LexA n°1 | est2 nup1-LexA n°13 | est2 rad52 n°11 | est2 rad52 n°32 | est2 rad52 n°53 | est2 rad52 n°1 | est2 rad52 nup1-LexA n°8 | est2 rad52 nup1-LexA n°12 | est2 rad52 nup1-LexA n°17 | est2 rad52 nup1-LexA n°23 | est2 rad52 nup1-LexA n°34 | est2 rad52 nup1-LexA n°37 | est2 rad52 nup1-LEXA n°49 | est2 rad52 nup1-LEXA n°70 |
|---|---|---|---|---|---|---|---|---|---|---|---|---|---|---|---|---|---|---|---|---|---|---|---|---|Cell density (OD600)
Time (days)

## Slide 3
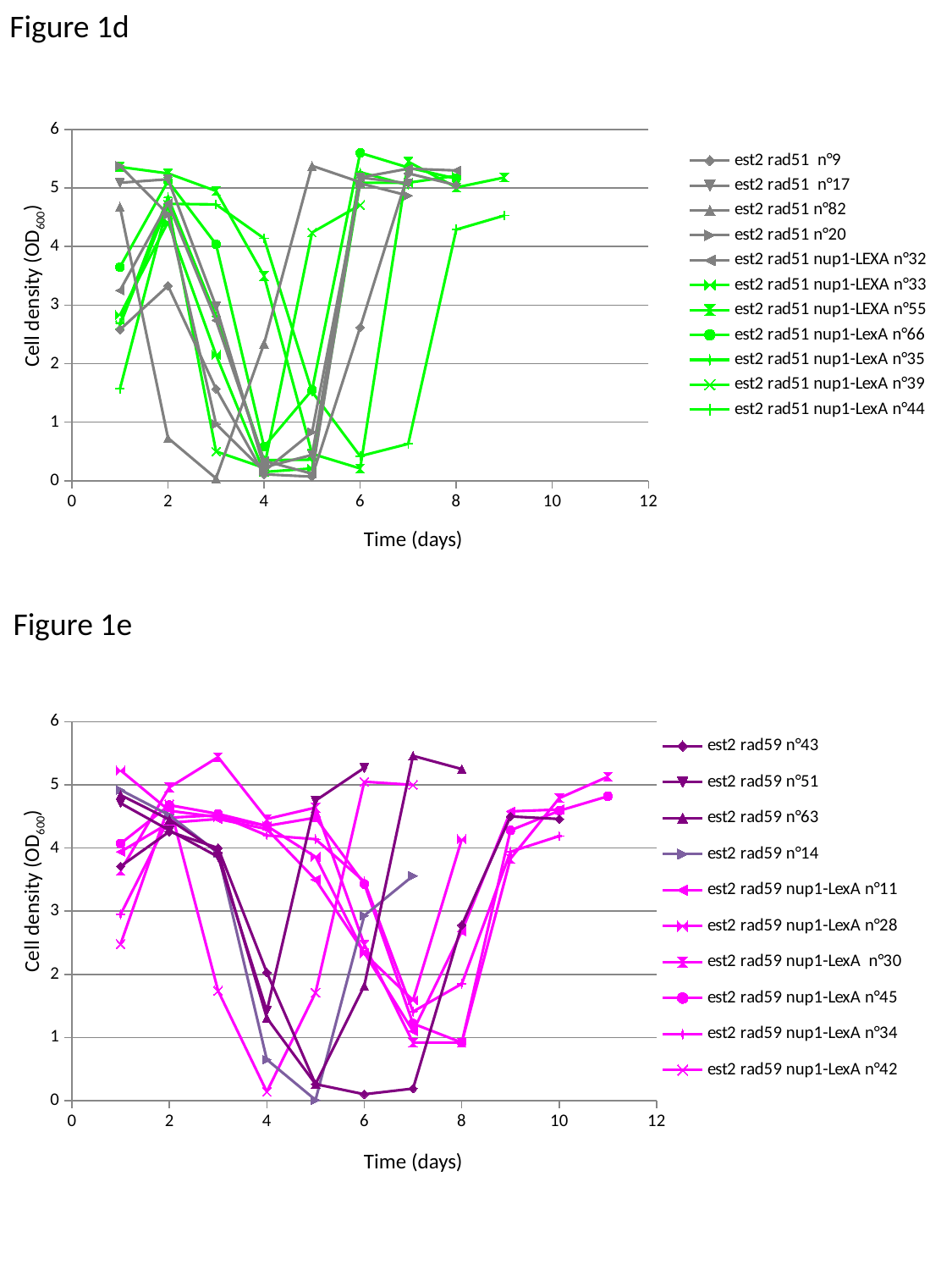

Figure 1d
### Chart
| Category | est2 rad51 n°9 | est2 rad51 n°17 | est2 rad51 n°82 | est2 rad51 n°20 | est2 rad51 nup1-LEXA n°32 | est2 rad51 nup1-LEXA n°33 | est2 rad51 nup1-LEXA n°55 | est2 rad51 nup1-LexA n°66 | est2 rad51 nup1-LexA n°35 | est2 rad51 nup1-LexA n°39 | est2 rad51 nup1-LexA n°44 |
|---|---|---|---|---|---|---|---|---|---|---|---|Cell density (OD600)
Time (days)
Figure 1e
### Chart
| Category | est2 rad59 n°43 | est2 rad59 n°51 | est2 rad59 n°63 | est2 rad59 n°14 | est2 rad59 nup1-LexA n°11 | est2 rad59 nup1-LexA n°28 | est2 rad59 nup1-LexA n°30 | est2 rad59 nup1-LexA n°45 | est2 rad59 nup1-LexA n°34 | est2 rad59 nup1-LexA n°42 |
|---|---|---|---|---|---|---|---|---|---|---|Cell density (OD600)
Time (days)

## Slide 4
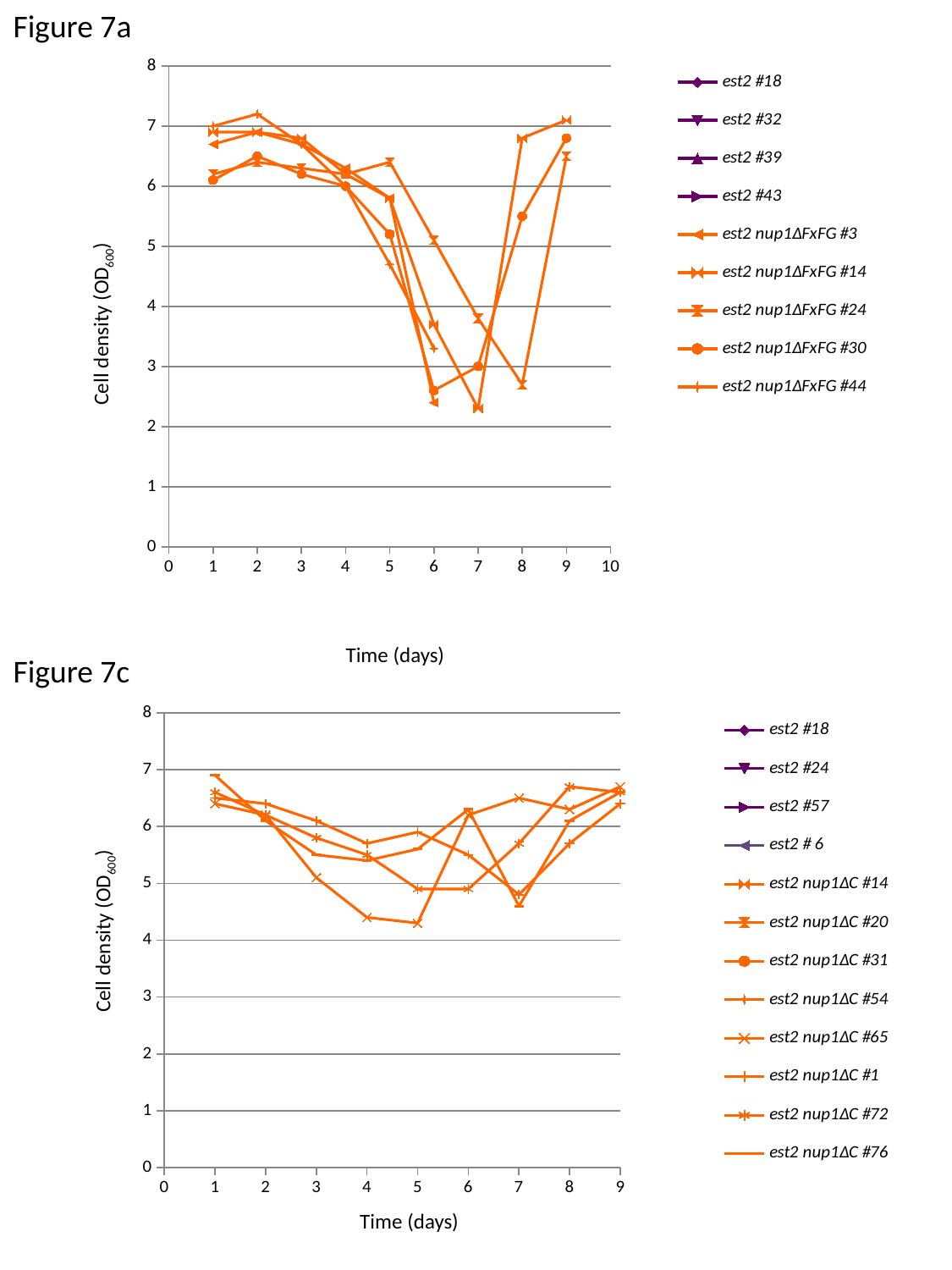

Figure 7a
### Chart
| Category | est2 #18 | est2 #32 | est2 #39 | est2 #43 | est2 nup1∆FxFG #3 | est2 nup1∆FxFG #14 | est2 nup1∆FxFG #24 | est2 nup1∆FxFG #30 | est2 nup1∆FxFG #44 |
|---|---|---|---|---|---|---|---|---|---|Cell density (OD600)
Time (days)
Figure 7c
### Chart
| Category | est2 #18 | est2 #24 | #REF! | est2 #57 | est2 # 6 | est2 nup1∆C #14 | est2 nup1∆C #20 | est2 nup1∆C #31 | est2 nup1∆C #54 | est2 nup1∆C #65 | est2 nup1∆C #1 | est2 nup1∆C #72 | est2 nup1∆C #76 |
|---|---|---|---|---|---|---|---|---|---|---|---|---|---|Cell density (OD600)
Time (days)

## Slide 5
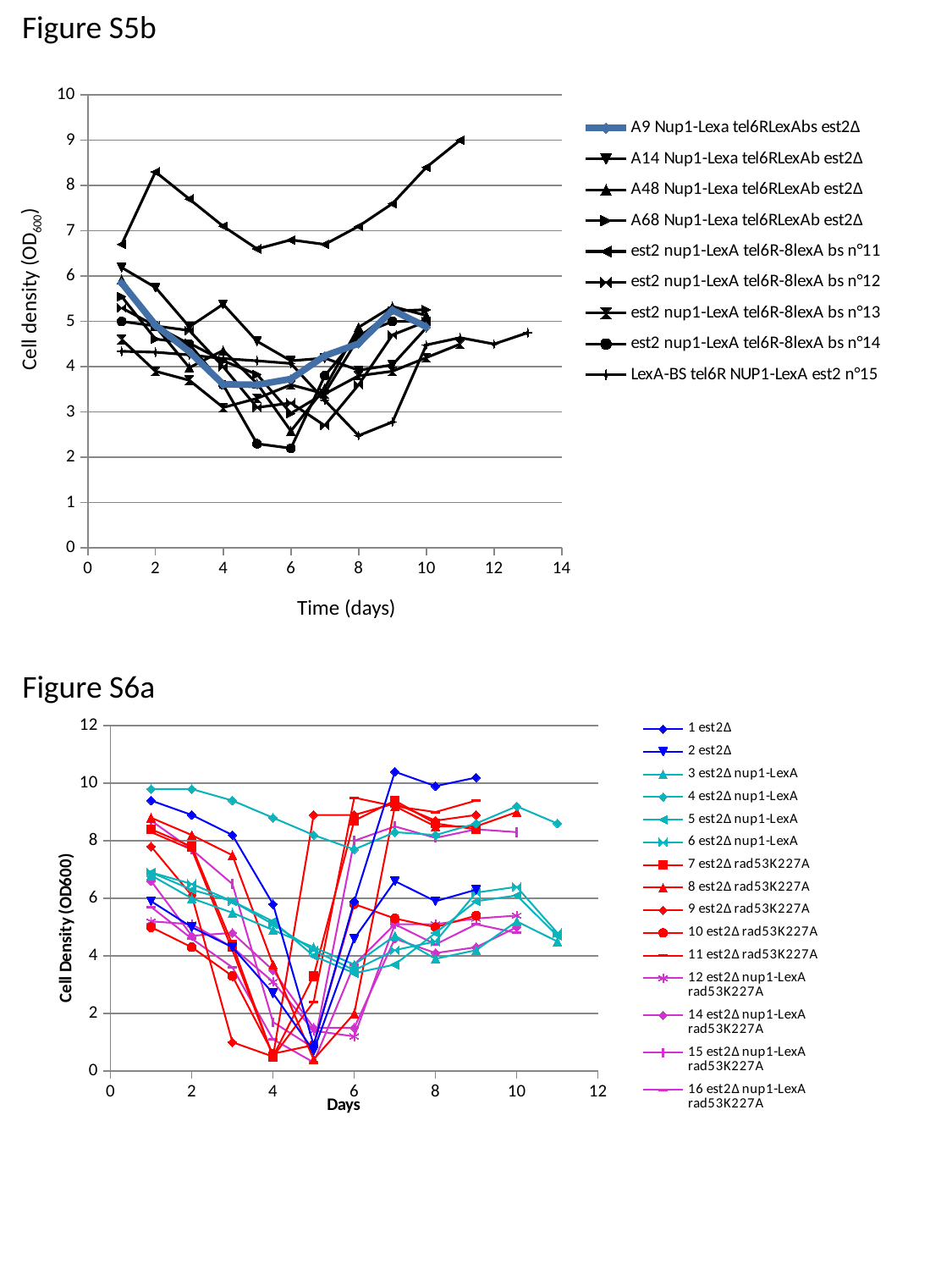

Figure S5b
### Chart
| Category | A9 Nup1-Lexa tel6RLexAbs est2∆ | A14 Nup1-Lexa tel6RLexAb est2∆ | A48 Nup1-Lexa tel6RLexAb est2∆ | A68 Nup1-Lexa tel6RLexAb est2∆ | est2 nup1-LexA tel6R-8lexA bs n°11 | est2 nup1-LexA tel6R-8lexA bs n°12 | est2 nup1-LexA tel6R-8lexA bs n°13 | est2 nup1-LexA tel6R-8lexA bs n°14 | LexA-BS tel6R NUP1-LexA est2 n°15 |
|---|---|---|---|---|---|---|---|---|---|Cell density (OD600)
Time (days)
Figure S6a
### Chart
| Category | 1 est2Δ | 2 est2Δ | 3 est2Δ nup1-LexA | 4 est2Δ nup1-LexA | 5 est2Δ nup1-LexA | 6 est2Δ nup1-LexA | 7 est2Δ rad53K227A | 8 est2Δ rad53K227A | 9 est2Δ rad53K227A | 10 est2Δ rad53K227A | 11 est2Δ rad53K227A | 12 est2Δ nup1-LexA rad53K227A | 14 est2Δ nup1-LexA rad53K227A | 15 est2Δ nup1-LexA rad53K227A | 16 est2Δ nup1-LexA rad53K227A |
|---|---|---|---|---|---|---|---|---|---|---|---|---|---|---|---|

## Slide 6
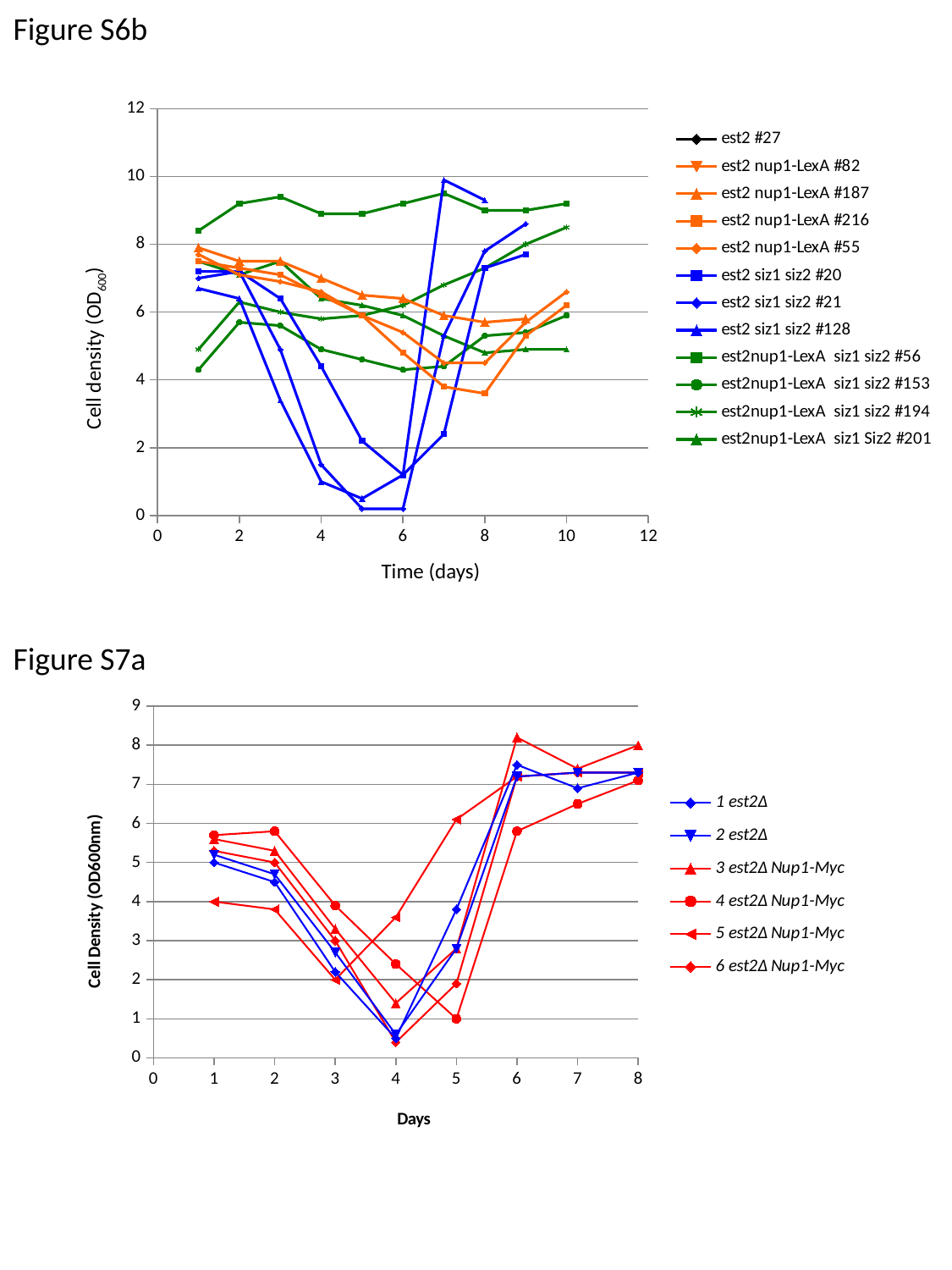

Figure S6b
### Chart
| Category | est2 #27 | est2 nup1-LexA #82 | est2 nup1-LexA #187 | est2 nup1-LexA #216 | est2 nup1-LexA #55 | est2 siz1 siz2 #20 | est2 siz1 siz2 #21 | est2 siz1 siz2 #128 | est2nup1-LexA siz1 siz2 #56 | est2nup1-LexA siz1 siz2 #153 | est2nup1-LexA siz1 siz2 #194 | est2nup1-LexA siz1 Siz2 #201 |
|---|---|---|---|---|---|---|---|---|---|---|---|---|Cell density (OD600)
Time (days)
Figure S7a
### Chart
| Category | 1 est2Δ | 2 est2Δ | 3 est2Δ Nup1-Myc | 4 est2Δ Nup1-Myc | 5 est2Δ Nup1-Myc | 6 est2Δ Nup1-Myc |
|---|---|---|---|---|---|---|

## Slide 7
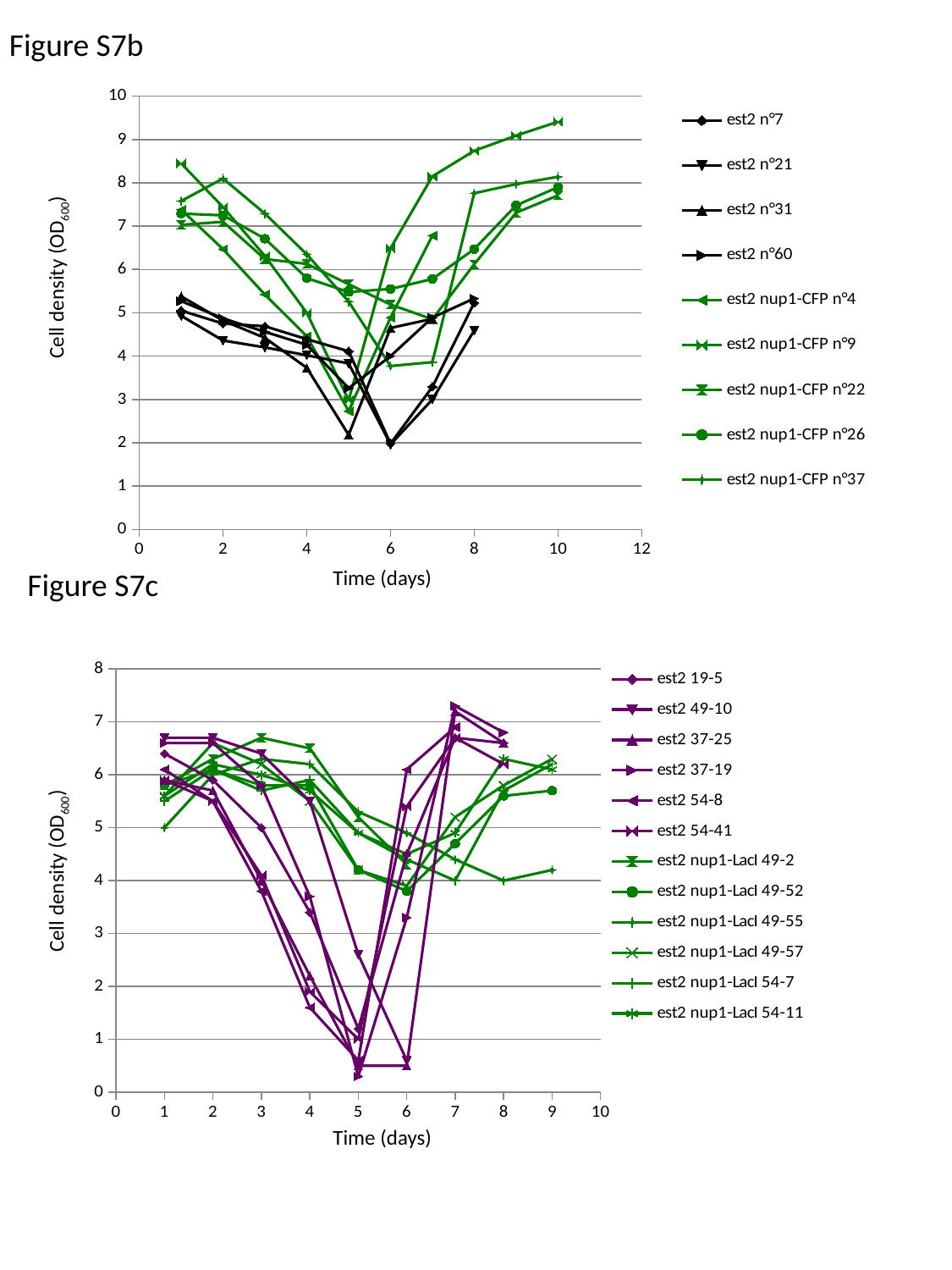

Figure S7b
### Chart
| Category | est2 n°7 | est2 n°21 | est2 n°31 | est2 n°60 | est2 nup1-CFP n°4 | est2 nup1-CFP n°9 | est2 nup1-CFP n°22 | est2 nup1-CFP n°26 | est2 nup1-CFP n°37 | | | |
|---|---|---|---|---|---|---|---|---|---|---|---|---|Cell density (OD600)
Figure S7c
Time (days)
### Chart
| Category | est2 19-5 | est2 49-10 | est2 37-25 | est2 37-19 | est2 54-8 | est2 54-41 | est2 nup1-LacI 49-2 | est2 nup1-LacI 49-52 | est2 nup1-LacI 49-55 | est2 nup1-LacI 49-57 | est2 nup1-LacI 54-7 | est2 nup1-LacI 54-11 |
|---|---|---|---|---|---|---|---|---|---|---|---|---|Cell density (OD600)
Time (days)

## Slide 8
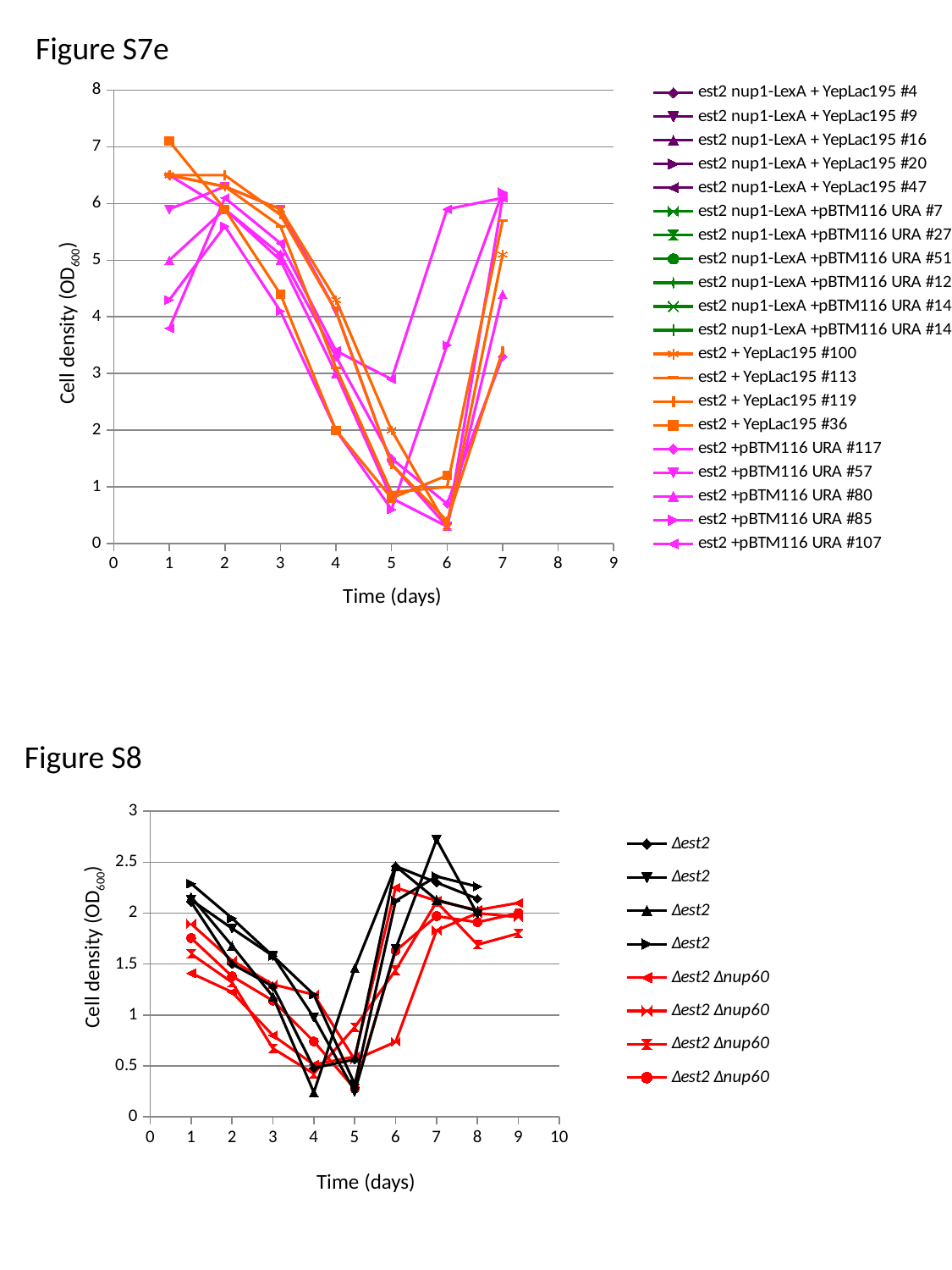

Figure S7e
### Chart
| Category | est2 nup1-LexA + YepLac195 #4 | est2 nup1-LexA + YepLac195 #9 | est2 nup1-LexA + YepLac195 #16 | est2 nup1-LexA + YepLac195 #20 | est2 nup1-LexA + YepLac195 #47 | est2 nup1-LexA +pBTM116 URA #7 | est2 nup1-LexA +pBTM116 URA #27 | est2 nup1-LexA +pBTM116 URA #51 | est2 nup1-LexA +pBTM116 URA #123 | est2 nup1-LexA +pBTM116 URA #143 | est2 nup1-LexA +pBTM116 URA #145 | est2 + YepLac195 #100 | est2 + YepLac195 #113 | est2 + YepLac195 #119 | est2 + YepLac195 #36 | est2 +pBTM116 URA #117 | est2 +pBTM116 URA #57 | est2 +pBTM116 URA #80 | est2 +pBTM116 URA #85 | est2 +pBTM116 URA #107 |
|---|---|---|---|---|---|---|---|---|---|---|---|---|---|---|---|---|---|---|---|---|Cell density (OD600)
Time (days)
Figure S8
### Chart
| Category | Δest2 | Δest2 | Δest2 | Δest2 | Δest2 Δnup60 | Δest2 Δnup60 | Δest2 Δnup60 | Δest2 Δnup60 |
|---|---|---|---|---|---|---|---|---|Cell density (OD600)
Time (days)
